# Luminescence Enhancement Due to Symmetry Breaking in Doped Halide Perovskite Nanocrystals

**DOI:** 10.1021/jacs.2c07111

**Published:** 2022-08-17

**Authors:** Ghada H. Ahmed, Yun Liu, Ivona Bravić, Xejay Ng, Ina Heckelmann, Pournima Narayanan, Martin S. Fernández, Bartomeu Monserrat, Daniel N. Congreve, Sascha Feldmann

**Affiliations:** † Department of Electrical Engineering, Stanford University, Stanford, California 94305, United States; ‡ Cavendish Laboratory, University of Cambridge, Cambridge CB30HE, U.K.; § Department of Materials Science and Metallurgy, University of Cambridge, Cambridge CB30FS, U.K.; ∥ Rowland Institute, Harvard University, Cambridge, Massachusetts 02142, United States

## Abstract

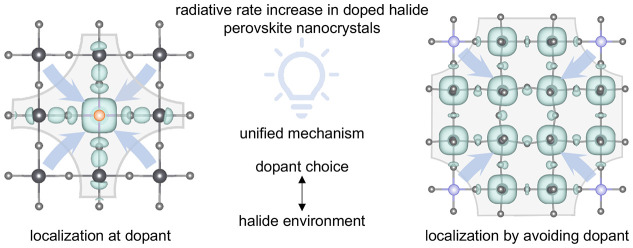

Metal-halide perovskite nanocrystals have demonstrated excellent optoelectronic properties for light-emitting applications. Isovalent doping with various metals (M^2+^) can be used to tailor and enhance their light emission. Although crucial to maximize performance, an understanding of the universal working mechanism for such doping is still missing. Here, we directly compare the optical properties of nanocrystals containing the most commonly employed dopants, fabricated under identical synthesis conditions. We show for the first time unambiguously, and supported by first-principles calculations and molecular orbital theory, that element-unspecific symmetry-breaking rather than element-specific electronic effects dominate these properties under device-relevant conditions. The impact of most dopants on the perovskite electronic structure is predominantly based on local lattice periodicity breaking and resulting charge carrier localization, leading to enhanced radiative recombination, while dopant-specific hybridization effects play a secondary role. Our results suggest specific guidelines for selecting a dopant to maximize the performance of perovskite emitters in the desired optoelectronic devices.

## Introduction

Nanocrystalline materials based on metal-halide perovskites like CsPbX_3_ (X = Cl, Br, I) have been shown to act as efficient solution-processable emitters for solid-state lighting and displays^1,2^ and are promising for emerging quantum light applications based on single-photon^3^ or spin-polarized emission.^4,5^ Atomic doping of these materials, mostly based on substitution of the Pb^2+^ ion for isovalent metal cations, has helped to further improve spectral tunability and photoluminescence quantum efficiency (PLQE), particularly in the blue spectrum where efficient light-emitting diodes (LEDs) remain a pressing goal.^6−9^ Recently, it was shown that for the case of manganese (Mn^2+^) doping the observed efficiency gains are the result of not only a reduction of non-radiative charge trapping but also of dopant-induced carrier localization, resulting in enhanced radiative recombination rates.^10^ However, a mechanism of how doping influences the optical properties in perovskite nanocrystals with other dopants is still missing and with it any generalizable understanding which captures all observed effects. However, it is this overarching mechanism that will be essential to maximize the performance of doped perovskite nanocrystals and guide the informed choice of doping element for device applications on a case-by-case basis.

For the first time, we directly measure, model, and compare the optical properties of the most effective doped perovskite nanocrystal compositions currently employed (Mn^2+^, Ni^2+^, and Zn^2+^, for Cl^–^ and Br^–^ halide environments, and further all alkaline earth metals), fabricated under identical synthesis conditions. We can thus largely exclude any deviations between chemical compositions and experimental measurement artifacts, which are often inevitable when comparing results from different labs. We find that the observed properties of all doped systems investigated can be well understood via a delicate interplay of largely dopant-independent structural effects resulting from lattice periodicity breaking which induce band gap widening and a radiative rate increase on the one hand, and dopant-dependent chemical and electronic effects arising from orbital hybridization on the other hand. Our mechanistic insights provide direct guidelines for the design of the most efficient emitters for a given device application by exploiting the synthetically accessible chemical space.

## Results

**Figure 1:**
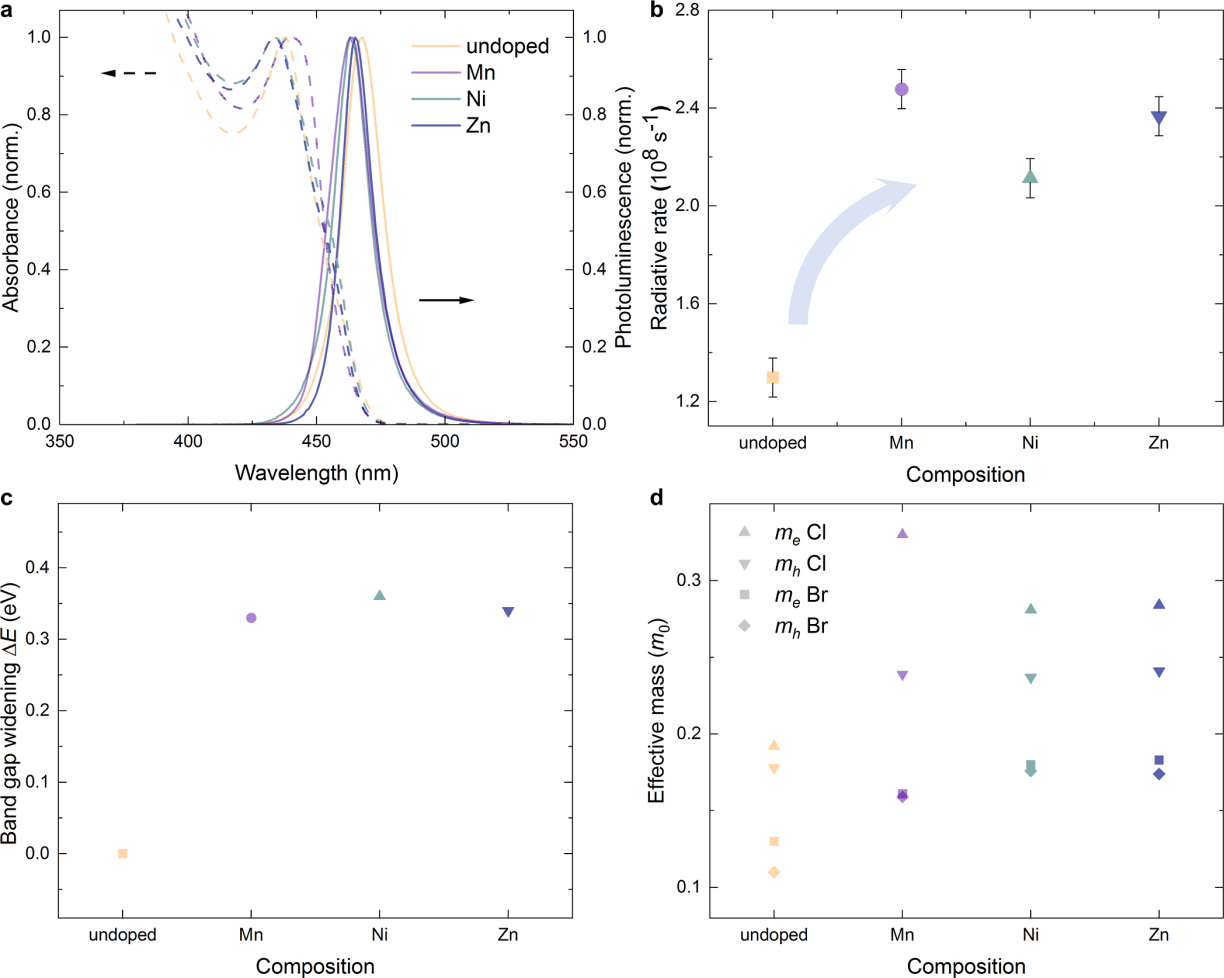
Doping-enhanced optoelectronic properties of perovskite nanocrystals. (a) Absorbance (dashed) and photoluminescence (PL) spectra (continuous lines) of undoped and doped CsPbCl_X_Br_3–*x*_ NCs, chemically calibrated to have a similar emission wavelength. Doping concentration is 1.5 ± 0.5 atomic-%. (b) Radiative recombination rates extracted from PLQE and time-resolved PL data (see the Supporting Information for details). The Mn^2+^ rate is extrapolated to correspond to the same concentration as for the other dopants. All samples were photoexcited at 405 nm. (c) Calculated dopant-induced electronic band gap widening without synthetic tuning through the halide composition (see the Methods Section in the Supporting Information for details). (d) Calculated effective masses of different dopant-halide systems, increasing upon doping and reproducing all measured trends remarkably well.

A series of the most commonly employed doped perovskite nanocrystals based on the transition-metal ions Mn^2+^, Ni^2+^, and Zn^2+^ isovalent to Pb^2+^ was synthesized (see the Supporting Information for details) and their steady-state absorbance and photoluminescence were characterized (Figure 1a). Further below, we show that the same effects can also explain doping with, for example, alkaline-earth metals (see Supporting Information, Figures S7–S10 for a detailed investigation on those). The fact that we study here dopants with the same oxidation state as the Pb^2+^ ion in undoped nanocrystals also means we can separate the dopant effects discussed below from those arising from p- or n-type charge doping effecting carrier populations and dynamics.

We study here doping concentrations of 1–2 atomic-% because these have demonstrated the highest PLQE (Supporting Information, Figure S5), while higher concentrations overly distort the perovskite host structure and induce additional non-radiative trap channels. Importantly, doping has been reported to induce a blue shift of the optical band gap in these materials.^7^ To decouple the dopant-induced changes to the radiative rate due to lattice periodicity breaking from those purely originating from a rate increase induced by the blue-shifted band gap, we synthetically calibrated all doped materials in such a way that they display very similar optical band gaps compared to the undoped material.

For this, we first dope the perovskite with the respective dopant ion, which consequently blue-shifted the observed emission. In order to spectrally calibrate all samples to have a very similar emission wavelength, we added a few mL of lead bromide stock solution to each composition until the emission maximum was red-shifted back to 470 nm. We chose an emission of approximately 470 nm because this corresponds to an essential color coordinate for blue LEDs in display applications, as the *y*-value 0.08 on the Commission International de l’Eclairage1931 (CIE 1931) chromaticity diagram forms the primary blue standard according to the National Television System Committee.^11^ The doping concentration of the final resulting NCs was determined with inductively-coupled plasma mass spectrometry to be 1.5 ± 0.5 atomic-%, respectively (see the Supporting Information). All studied nanocrystals were measured to be dopant-independent cubic shaped with an average size of 10 ± 2 nm (see the Supporting Information Figure S1 for transmission electron microscopy images), implying the charge dynamics are situated within the typical weak quantum confinement regime,^12^ as is also confirmed by a linear PL dependence on excitation fluence (confirming excitonic recombination, see Supporting Information, Figure S2). Thus, no changes to the dielectric environment by NC shape or size could obscure the conclusions drawn from doping-induced changes to the optoelectronic properties, which we consequently investigate.

By measuring both the PLQE and the time-resolved PL decay of the nanocrystals, we readily quantify and compare the radiative recombination rate for each composition (Figure 1b, see the Supporting Information for details on the calculations and Figures S3–S5 for underlying data). We follow here the analysis from Klimov^12,13^ and other groups studying semiconductor nanocrystals: under the assumption that the measured NC ensemble is characterized by the same material-specific radiative rate—not influenced by changes to the dielectric screening, as we ensured through the monodispersity of size and shape of our NCs from TEM analysis, Supporting Information, Figure S1—the multiexponential PL decay observed relates to a variety of individual non-radiative rates differing from NC to NC due to, for example, the difference in the number and/or nature of centers of non-radiative recombination (traps). As such, the radiative rate can be extracted from the PLQE of the ensemble together with their average total exciton lifetime measured as the PL decay, accounting for all radiative and non-radiative processes (see the Supporting Information for details). We note that this analysis would not be valid for perovskite materials with charge dynamics governed by free charges showing complex trapping and de-trapping behavior.^14,15^ Importantly, for all doped compositions, we find a—now band gap-unrelated, as spectrally calibrated—substantial radiative rate increase of 63–92% compared to the undoped NCs. This generalizes the initial observation made for Mn-doping before,^10^ where we showed that this rate increase directly relates to an increase in the oscillator strength of the electronic transition (see Supporting Information, Figure S6 for oscillator strength values). We stress that these findings are distinctly different from the limited observation of increasing PLQEs upon doping, which is mostly assigned to trap-passivation in the literature, for example, by filling halide vacancies.^7^ Such reduction of non-radiative rates can be achieved by external changes to the semiconductor, for example, by surface passivation or stability approaches, and can dramatically improve PLQEs.^16,17^ It leaves, however, the highest achievable light emission for a given semiconductor untouched, as the intrinsic radiative rate remains unchanged in this case, and comes with drawbacks for devices, for example, through unfavorable work function shifts,^18^ or reduced carrier mobilities if long insulating ligands are employed. Moreover, PLQEs vary largely between different synthesis conditions for seemingly identical doped materials, as well as between different labs, because of the sensitivity of PLQE to non-radiative losses. While we do indeed observe strong PLQE increases and show that they are also concomitant with a reduction in the non-radiative recombination rate (see Supporting Information, Figure S5), it is thus the doping-induced increase of the intrinsic radiative rate that is most remarkable and more fundamental to compare here. It is also unaffected by the varying (trap-density-convoluted) PLQE values reported for identical compositions by different labs. Aside from allowing operation at higher maximum brightness for LED applications in lighting and displays for a given semiconductor, an increased radiative rate will also enable important technologies based on quantum coherent phenomena, like those relying on lasing,^19^ single-photon emitters,^3^ or superfluorescence.^20,21^

We rationalize these experimental observations by performing electronic structure calculations using density functional theory (DFT, see the Supporting Information for details). We model the undoped and doped CsPbX_3_ systems for different halide environments (@X = Cl, Br) using 3 × 3 × 3 supercells, corresponding to nominal doping concentrations of 3.7%, to accurately reflect the low doping concentrations in the synthesized materials.

In Figure 1c, we show that the optical band gap increases upon doping compared to the pristine perovskite. In a previous study, we showed this result for the specific case of Mn doping and could relate it to the concept of local perovskite lattice periodicity breaking through the dopant.^10^ Here, we expand this observation to be valid also for all other doped systems studied and explain how any dopant will exhibit the same effect. Moreover, as shown experimentally above (Figure 1b), the electron–hole overlap increases upon doping and with it the radiative recombination rate—an intrinsic property that is very hard to influence generally for a given semiconductor. Within our theoretical framework, this can be approximately tracked as the effective carrier mass of the electronic transition (Figure 1d). We find that the effective mass increases significantly for all studied dopants, by 48–76% compared to the undoped system, and reproduces the measured radiative rate trends remarkably well. In detail, the observed increase for a chloride environment (@Cl) is the strongest for Mn, followed by Zn and, only slightly smaller, Ni doping. The electron effective mass exhibits larger increases for the Mn@Cl case than for the other dopants, whereas for the bromide environment (@Br), Zn doping shows the largest effective mass increase.

**Figure 2:**
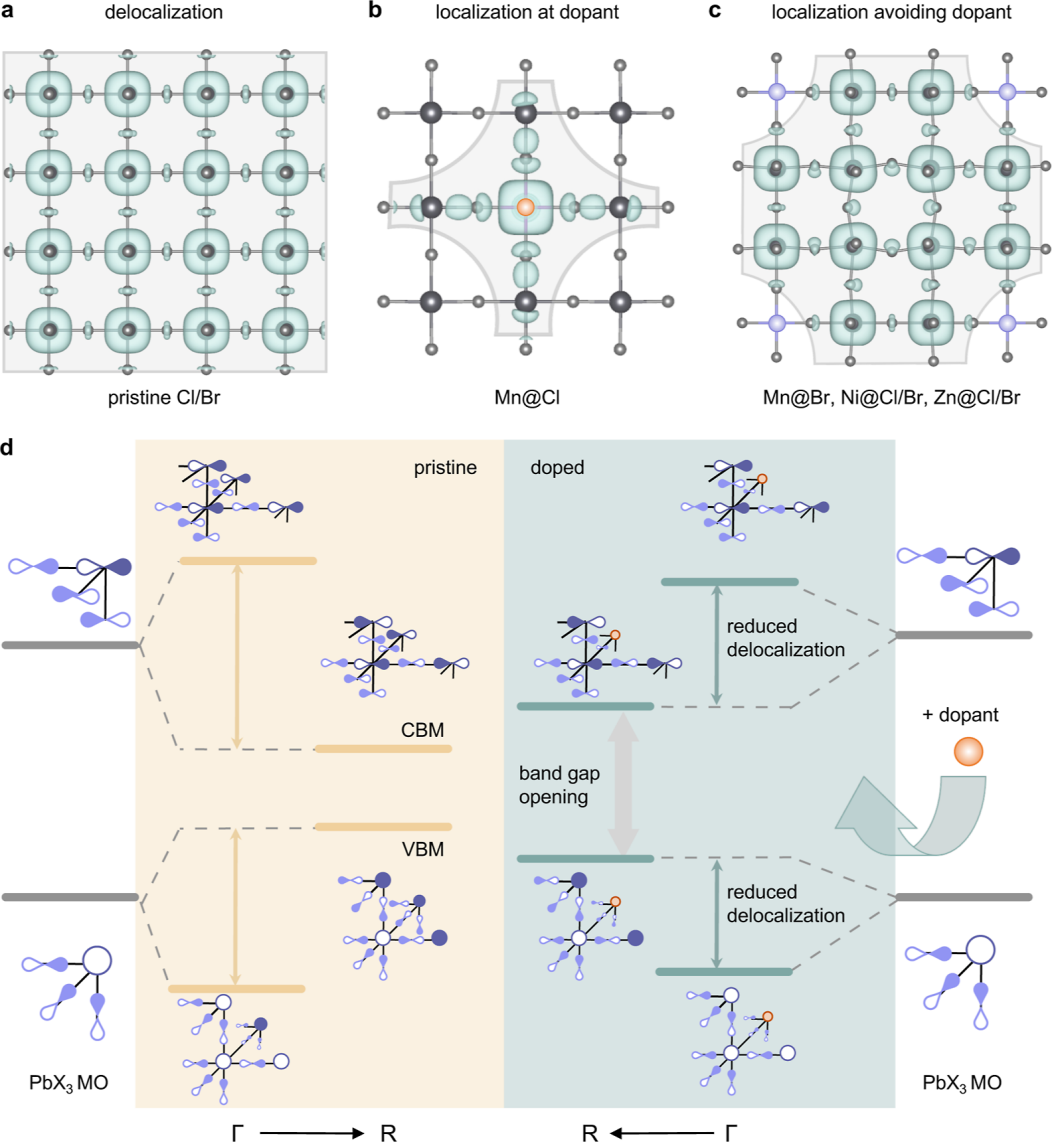
Dopant- and halide environment-dependent localization mechanism in perovskite nanocrystals. (a) Isosurface of the conduction band minimum electron density (green), delocalized across the lattice (grey) in undoped perovskite NCs. (b) Charge localization induced by the dopant in the special case of Mn (orange) in a chloride environment, where hybridization occurs. Value at electron density isosurface is 2.36 × 10^–3^ e Å^–3^. (c) Reduced charge delocalization through dopant potential well formation in the absence of hybridization, as is the case for all other dopant–halide systems studied (here shown for the example of Zn, blue, in a chloride environment). All cases result in faster radiative rates. Results based on 3 × 3 × 3 supercells. (d) Underlying orbital model. Left: schematic representation of the Bloch states formed from the perovskite host PbX_3_ molecular orbitals (MOs) at the Γ-point and the R-point, contributing to the valence and the conduction bands, respectively. The yellow double arrows indicate the bandwidth of the bands. Right: representation of the same states upon incorporation of any B-site dopant (orange). It becomes clear that (i) the band gap widens because of stabilization and destabilization of the VBM and the CBM, respectively (see grey double arrow), and (ii) that the bandwidth for both valence and conduction bands is reduced upon doping (green double arrows), resulting in higher effective masses and radiative rates.

We now rationalize all these observations, both experimentally and from a high level of theory, in a unified approach based on orbital symmetries, and demonstrate its implications for the charge carrier distribution in a real space for the different cases, as shown in Figure 2.

We show that the dominant driving force for the band gap increase as well as, importantly, the effective mass and radiative rate gains in all systems is the doping-induced lattice periodicity breaking, which is element unspecific. Therefore, every doped material (Figure 2b,c) exhibits a distortion in the charge density distribution compared to the homogeneously delocalized one observed for the pristine perovskite (Figure 2a). We note that, while the finite size of our nanocrystals already breaks to a certain degree the (infinite) lattice periodicity assumed in an electronic band structure model, this quantum size effect is expected to be the same across all undoped and doped compositions, as we do not observe changes to the shape or size of the NCs. Thus, we can treat the doping-induced periodicity breaking effect independently, and in addition to the surface effect present throughout. Following the orbital model that has been proposed by Goesten and Hofmann^22^ for pristine perovskites before, the cubic perovskite conduction band is constructed by an antibonding atomic PbX_3_ basis (Figure 2d, see Supporting Information and Figures S11–13 for a more detailed discussion). To obtain delocalized bands, a phase factor is introduced at different wave vectors *k* in the electronic Brillouin Zone (BZ). At the *R*-point [*k* = (1/2, 1/2, 1/2)], the Bloch state exhibits a phase change between each neighboring antibonding PbX_3_ orbital basis, creating a bonding interaction between them (see also Supporting Information Figures S6 and S7) and consequently stabilizing the electronic state at the *R*-point [thus becoming the conduction band minimum (CBM)]. This universally explains the blue shift upon doping.

To explain the increase in effective masses (and radiative rates) observed for all dopants, we also need to consider the electronic CB states toward the BZ center (*k* = 0), that is, not only at the R-point but also at the Γ-point. At Γ, the electronic band does not exhibit a phase change between the neighboring PbX_3_ bases, thus resulting in a destabilizing antibonding interaction between each site, in contrast to the bonding interaction at the R-point. Consequently, B-site doping leads to the opposite effect in the zone center compared to the zone boundary and stabilizes the conduction band toward Γ. An analogous argument leads to the converse effect for the valence band. Therefore, the simultaneous destabilization at the R-point and the stabilization at the Γ-point upon doping ultimately reduces the bandwidth and dispersion of both conduction and valence bands.

This reduced dispersion manifests itself in real space as a reduced delocalization of the charge density with charge depletion at the B-site dopant, which now acts as a potential well (Figure 2c), resulting in increased effective masses and radiative rates, as we observe. As such, the electronic response is dictated by the symmetry of the atomic bases building the band edge states and is therefore universal for all doped cubic lead-halide perovskites. To demonstrate this, we extended our analysis to the chemically dissimilar alkaline earth metals and present an extensive study in the Supporting Information (Supporting Information, Figures S8–S11), confirming the effect of lattice periodicity breaking is largely independent of the electronic configuration of the perturbing dopant element and mostly depending on its size (i.e., Goldschmidt factor, Supporting Information, Figure S9) and concentration (i.e., amount of perturbance, Supporting Information, Figure S10).

We also confirm the role of the electronic effect by which a dopant can affect the charge density distribution. This effect is element specific and is found to be significant only in the case of manganese and only in a chloride environment (Figure 2b). Here, the s orbitals do match well energetically and symmetry-wise with the perovskite conduction band edge and thus show a significant degree of hybridization, absent in all other studied systems. These newly formed hybridized states are lower in energy than the pristine perovskite and therefore lead to a localization around the dopant, thus strongly enhancing the radiative rates. While Mn@Cl therefore provides a special case within the studied systems with respect to the mechanism, that is, charge localization toward the dopant rather than away from it, as is the case for all other systems studied, the resulting reduced spread of the Bloch waves and concomitantly observed radiative rate increase is very similar.

**Figure 3:**
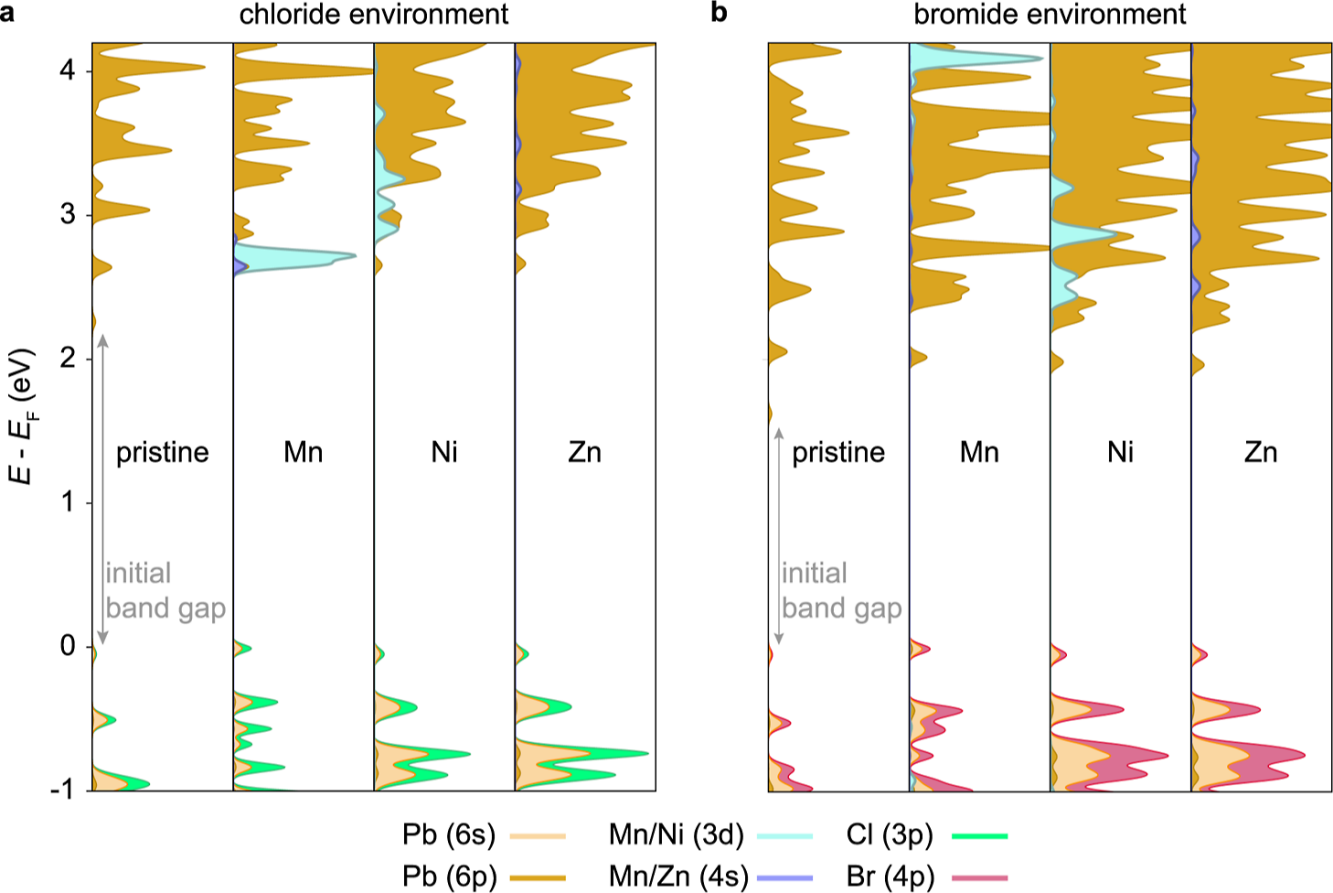
Projected density of states (pDoS) of pristine and doped halide perovskites. (a) Dopant-dependent pDoS for a chloride environment, where only the Mn 4s orbitals can hybridize with the perovskite conduction band edge states, leading to an enhanced localization effect (see the Supporting Information for details). (b) Dopant-dependent pDoS for a bromide environment where no dopant hybridization occurs. Gray arrows indicate the initial band gap of the pristine systems before doping-induced widening occurs. Results based on 3 × 3 × 3 supercells.

However, manganese in a chloride-rich environment is also the only doped system where the dopant d orbitals lie energetically within the perovskite band gap, as becomes evident in the project density of states for the different dopants (Figure 3).

For Mn, these d states do not hybridize with the perovskite host band edges and instead act as the commonly observed energy loss channel for excitations, resulting in a spin- and symmetry–forbidden transition. This is commonly observed upon a threshold Mn doping concentration as a long-lived orange (∼600 nm) emission.^23,24^ It limits the application of manganese doping to achieve color-pure and bright blue emission in LEDs: a maximal doping concentration of about 0.2% (Mn/Pb atomic ratio)^10^ exploits the doping-induced radiative rate benefits without yet forming those additional loss channels in significant amounts which overall reduce the PLQE of the blue perovskite emission beyond this concentration.

In contrast to manganese in a chloride environment, for the other systems the dopant d orbitals lie energetically higher within the perovskite conduction bands and thus cannot act as such a loss channel. For Ni, there is, however, a significant DoS for the d orbitals present, which partially screens charges and thus reduces the electron–hole attraction that leads to radiative recombination. Zinc with its closed d shell instead shows no significant density of d-orbital states and thus displays a slightly larger radiative rate compared to nickel (the same holds true for the closed-shell alkaline earth elements), as electron–electron correlations are less pronounced, and as is also confirmed experimentally (Figure 1). For a bromide-rich environment, the energy levels are shifting such that Mn s orbital hybridization becomes negligible and the additional electronic effect compared to the other dopants vanishes, as does the orange d-band emission. These findings have a profound impact on the practical guidance of dopant choice for applications, as we will discuss below. As the above symmetry arguments should in principle remain valid for unconfined bulk halide perovskites as well, it would be promising to study such systems in the future, if successful incorporation of isovalent dopants into the lattice can be achieved.

**Figure 4:**
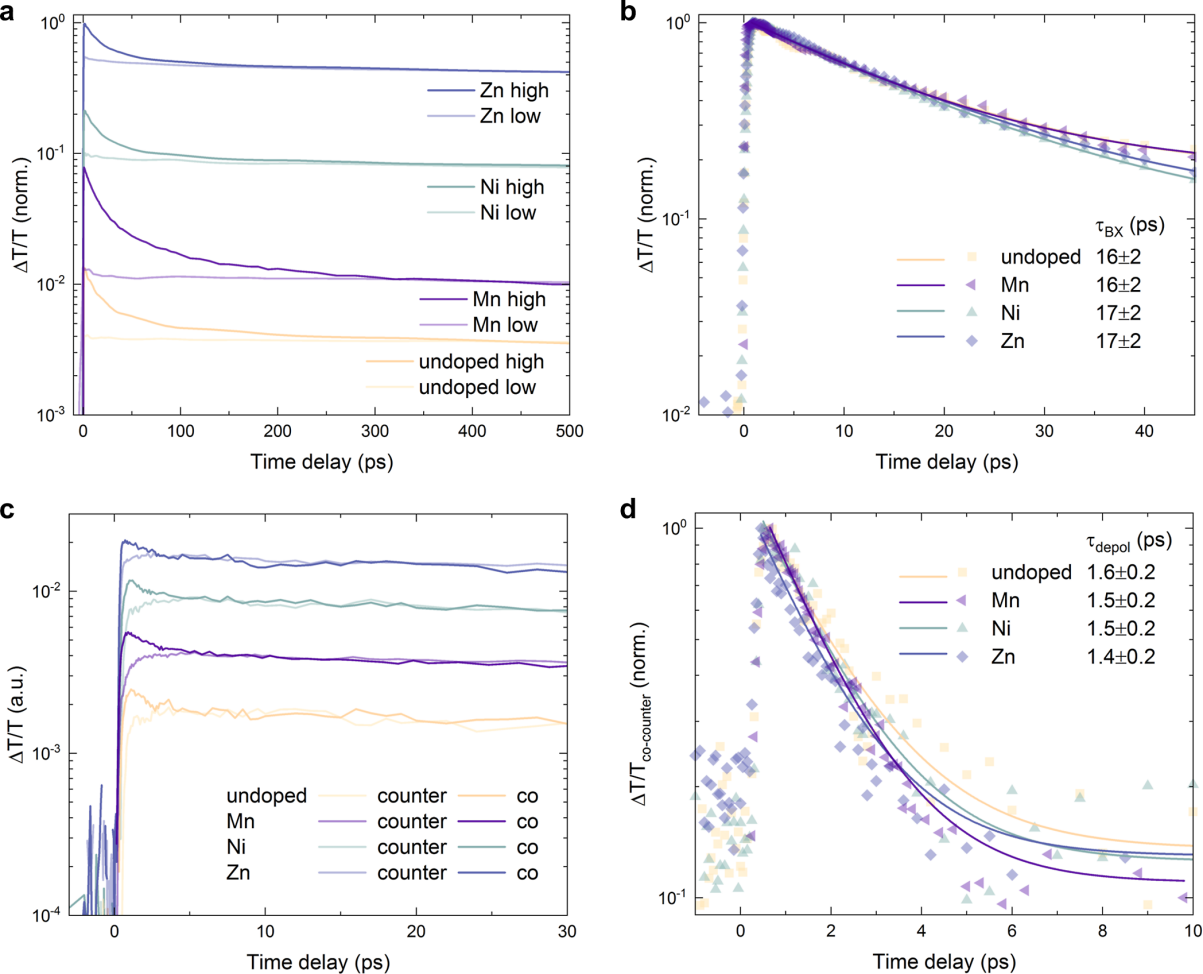
Doping does not affect biexciton and spin lifetimes in NCs under device-relevant conditions. (a) High excitation-fluence (dark curves, ⟨*N*⟩_0_ = 31) and low-fluence (light curves, ⟨*N*⟩_0_ = 0.1) ground-state bleach kinetics extracted from transient absorption (TA) data, vertically off-set for clarity. (b) Monoexponential fits to the kinetics obtained by subtracting the low- from the high-fluence data in (a). Very similar biexciton lifetimes are extracted for all compositions. (c) Co-polarized (dark curves) and counter-polarized (light curves) ground-state bleach kinetics extracted from TA data (fluence ⟨*N*⟩_0_ = 0.3, respectively), vertically off-set for clarity. (d) Monoexponential fits to the kinetics obtained by subtracting the co-from the counter-polarized data in (c). Very similar spin-depolarization lifetimes are extracted for all compositions. All samples were photoexcited at 400 nm (∼100 fs pulses, repetition rate 1 kHz) and measurements performed at room temperature.

Lastly, we also performed ultrafast transient absorption (TA) spectroscopy to rule out other, more exotic doping-induced effects that one might hypothesize could play a significant role in the optoelectronic properties of these systems under device-relevant conditions and which would impact the choice of dopants; specifically, multi-exciton and spin-related phenomena taking place at ultrafast time scales (Figure 4).

For extracting bi-exciton lifetimes, we first compare the ultrafast transient absorption kinetics at a very low and a very high excitation density (Figure 4a, ⟨*N*⟩_0_ is the initial average excitation density per NC, see the Supporting Information for details). At high laser fluence, the formation of multi-exciton species can be observed, which can lead to altered carrier dynamics and emission properties as discussed in the literature.^13,25,26^ By subtracting the late-time normalized low- from the high-fluence data, the multiexciton recombination rate can be estimated from a monoexponential fit to the resulting early-time kinetics as shown before by Klimov and others^13^ (Figure 4b). We observe very similar multiexciton (i.e., most likely biexciton) recombination times of about τ_BX_ = 16 ± 2 ps for all the studied doping systems and therefore rule out any significant doping-induced modification of multi-exciton dynamics.

Finally, to determine the spin-relaxation times, we studied the compositions using circularly polarized transient absorption spectroscopy (Figure 4c,d). Because some of the transition-metal dopants possess a net magnetic moment due to their unpaired d-electrons, one might expect an influence on the spin-dynamics in the doped perovskite systems.^27^ We compare the co- and counter-polarized kinetics and (Figure 4c) and subtract them to retrieve the spin-depolarization lifetime through a monoexponential fit of the resulting decay^4,27,28^ (Figure 4d). We find that this lifetime, at which an equilibrium between the different total angular momentum sub-states (*m*_J_ = −1/2 and +1/2) is reached, is very similar across all doped systems and estimated to be τ_depol_ = 1.5 ± 0.2 ps. We therefore also exclude any significant doping-induced influence on the spin dynamics of the perovskite with respect to the optoelectronic properties at room temperature and for our studied NC sizes, leaving the discussed charge localization effects as the only relevant ones that intrinsically impact the light emission under device-relevant conditions. While a recent report on the influence of nickel doping suggests a certain degree of spin-exchange coupling,^29^ those measurements relied on the presence of strong magnetic fields and low temperatures, making the phenomena conceptually interesting for spintronics applications but unlikely to impact the optoelectronic properties under device conditions, that is, at room temperature and zero field. We can thus now conclude with the following remarks for the informed choice of the dopant system.

## Guidelines Arising for Choice of Dopant Based on Application

It follows that, if the desired device emission is to be optimized for higher wavelengths, a bromide-rich halide environment is to be chosen over a chloride one due to the lower band gap of the former. Here, we recommend the choice of zinc as a dopant, as (i) no additional s-orbital hybridization could be exploited in this environment anyways, (ii) Zn^2+^ then shows the strongest dopant-induced lattice periodicity breaking effect for enhancing the radiative rate without the partial screening present for the open-shell configurations in Mn^2+^ and Ni^2+^, and (iii) it also avoids potential d orbital induced loss channels present in Mn^2+^. If the desired application instead calls for a broad, dual, or long-lived emission spectrum, manganese doping with its additional orange (600 nm) d-state emission should be chosen, which is also not present in the partially filled d orbitals for nickel, due to its energetic positions. If the device is instead meant to be used in spintronics applications, then both Mn and Ni could be potentially useful due to their unpaired spins. If the desired emission wavelength should be in the very blue spectral range instead, a chloride-rich halide environment is to be used in general. As such, in the low-doping regime, manganese is the optimal dopant, as its s orbital hybridization leads to the strongest carrier localization, boosting the radiative rate gains further (and more so than the partial d-electron screening would counteract this, also because the DoS here is sufficiently far away energetically from the perovskite band edges). In the high doping regime, Mn becomes, however, suboptimal due to the formed d-state loss channels for the blue host emission. Thus, Zn would in this regime be the best choice (over Ni for the above electron screening reasons), or indeed alkaline earth metals. However, Ni and Zn form also less stable compositions, as measured by the PLQE dropping by about 30% within 5 days outside an inert atmosphere, while instead Mn-doped NCs sustained their PLQE for more than 3 months outside an inert atmosphere. This superior stability of Mn-doped NCs may be rationalized by the unique electronic effect of orbital hybridization in this case, as discussed above. Here, the charges also become localized around the dopant for efficient radiative recombination (Figure 2b), and they are thus less likely to diffuse to the trap sites distributed across the remaining perovskite lattice. In contrast, for the other dopant systems, the charge density becomes localized in the perovskite host by avoidance of the perturbing dopant (i.e., a reduction in delocalization, see Figure 2c), and thus are more susceptible to the NC trap density. A balance between absolute performance gains and stability should thus be considered when choosing the correct dopant system for the desired application.

## Conclusions

In summary, we have experimentally and theoretically established a generalizable model for the working mechanism of doping in perovskite nanocrystals. We found this to be a largely dopant-independent lattice periodicity breaking effect increasing radiative rates, which is further modulated by more subtle effects like orbital hybridization, screening from electron–electron interactions and the respective halide environment. Our findings allow for the informed choice of the optimal doping system for a given optoelectronic device application.

## Supporting Information Available

Methods and experimental details, excitation power dependent photoluminescence, TEM images, time-resolved photoluminescence decays, discussion about other doping elements, band structures (PDF)
