# Supplementary material for: Luminescence Enhancement Due to Symmetry Breaking in Doped Halide Perovskite Nanocrystals
Source: J Am Chem Soc. 2022 Aug 17;144(34):15862–70. doi: 10.1021/jacs.2c07111 (PMC9437917; doi:10.1021/jacs.2c07111)
Supplement: Supplementary file 1 — ja2c07111_si_001.pdf [file ja2c07111_si_001.pdf]

Supplementary Information for

Luminescence enhancement due to symmetry breaking in doped halide perovskite nanocrystals

Ghada H. Ahmed[1], Yun Liu[2], Ivona Bravić[2], Xejay Ng[2], Ina Heckelmann[2], Pournima Narayanan[1], Martin S. Fernández[1], Bartomeu Monserrat[2,3], Daniel N. Congreve[1], Sascha Feldmann[2,4]*

[1]Department of Electrical Engineering, Stanford University, Stanford, CA 94305, USA

[2]Cavendish Laboratory, University of Cambridge, Cambridge, CB30HE, UK

[3]Department of Materials Science and Metallurgy, University of Cambridge, Cambridge, CB30FS, UK

[4]Rowland Institute, Harvard University, Cambridge, MA 02142, USA

**Email: sfeldmann@fas.harvard.edu*

Methods

Nanocrystal synthesis

Precursor materials:

Cesium carbonate (Cs_2CO_3, 99.995%, metal basis), 1-octadecene (ODE, technical grade 90%), Lead (II) chloride ($PbCl_2$, powder 98%), Lead (II) bromide ($PbBr_2$, powder 99.99%), Oleylamine (OAm, technical grade 70%), Oleic acid (OA, technical grade 70%), Trioctylphosphine (TOP, technical grade 90%), Nickel(II) chloride (anhydrous, 99.99%), Zinc (II) chloride (anhydrous, 99%), Hexanes (98%, pure) all purchased from Sigma-Aldrich. All chemicals were used as received without further purification.

Preparation of cesium-oleate precursor:

Typically, Cs_2CO_3 (0.814 g) and ODE (40 mL) were added into a 100 mL 3-neck round-bottomed flask and dried under vacuum at 120°C for 1 hour. After degassing, the solution was heated under N_2 atmosphere. Then, (2.5 ml) of oleic acid (OA) was subsequently injected into the solution mixture, and heated under N_2 at 150°C until Cs_2CO_3 was completely dissolved yielding a clear solution. Afterwards, the solution was cooled to room temperature for storage, and reheated to 100°C before use, as the cs-oleate precipitates out at room temperature.

Synthesis of undoped $CsPb(Cl/Br)_3$ NCs:

165 mg of $PbBr_2$ (0.450 mmol), 83.6 mg of $PbCl_2$ (0.301 mmol), 20 mL of octadecene, 2 mL of dried oleylamine, 2 mL of dried oleic acid, and 2 mL of trioctylphosphine were loaded into a 100-mL three-neck round-bottomed flask, dried under vacuum at 130°C for 45 min. The flask was then filled with N_2 and heated to 150°C for 10 minutes under rigorous stirring. Following that, the temperature was raised to 165°C under N_2 and kept at this temperature for 5 min. Then, 1.7 mL of pre-heated cesium oleate solution was quickly injected into the solution. After

60 seconds, the reaction was quenched by immediate immersion of the reaction flask into an ice-water bath.

Synthesis of Zn^{2+} and Ni^{2+} doped $CsPb(Cl/Br)_3$ NCs:

For a typical synthesis of doped $CsPb(Cl/Br)_3$ NCs, 0.21 mg (0.572 mmol) of lead (II) bromide, two different doping concentrations (0.051, 0.071, 0.081 and 0.091 mg) of $NiCl_2$ or $ZnCl_2$, and 0.080 mg $MnCl_2$ (for comparison purposes), 20 mL of octadecene, 2 mL of dried oleylamine, 2 mL of dried oleic acid, and 2 mL of trioctylphosphine into a 100-mL three-neck round-bottomed flask. This was dried at 130 °C for 45 min and heated to 150°C under vacuum for 10 minutes. The yielded solution was then heated to 165°C under N_2 protection for 5 minutes, after which 1.7 mL of pre-heated Cs-oleate precursors was rapidly injected into the solution. After having reacted for 60 s, a crude product was cooled to room temperature in an ice-water bath.

Post-synthetic halide exchange:

A blue shifted PL emission was observed after doping the NCs with both Zn^{2+} and Ni^{2+} ions as compared to the undoped ones, and it was more prominent for the Ni^{2+} doped samples. Therefore, for comparison purposes, the PL was aligned back to 470 nm for all the NCs. The as-synthesized NCs crude solution was aligned by exchanging the nanocrystal solution with (1-5 ml) of $PbBr_2$ stock solution prepared as below:

2.2 g of $PbBr_2$ (6 mmol), 50 mL of octadecene, 2 mL of dried oleylamine, and 2 mL of dried oleic acid in a 100-mL round-bottomed flask at 130°C were all mixed and stirred under vacuum for 40 min, after which it was cooled down to room temperature. The amount of $PbBr_2$ stock solution added to the crude solution was carefully monitored by tracking the PL peak position before purifying or washing the nanocrystals.

Isolation and purification of the NCs:

After the crude solution was cooled with an ice-water bath, the aggregated NCs were separated by centrifuging for 5 min at 12000 rpm. Then, the NCs were redispersed in 6 mL of hexane and centrifuged again for 5 min at 12000 rpm, and the supernatant was discarded. After repeating the previous step one more time, the final precipitate was redispersed in 6 mL of hexanes. It should be noted that different isolation and purification protocols have been tested and the PLQE was carefully monitored. For these samples, it was found that adding antisolvent (*i.e.* anhydrous ethyl acetate or methyl acetate) quenches the PLQE for both the Zn and Ni doped samples.

Note on the halide treatment of the NCs:

The post-synthetic surface treatment we employ here works by adding an excessive amount of salts (mainly lead-based halides) to reconstruct the NC surface after washing with antisolvents. This will allow for passivating surface trap states and improve the PLQE through reduction of non-radiative losses. In our case, however, we tune the PL position by post-synthetic anion exchange through adding $PbBr_2$ salt to the as-synthesized colloidal nanocrystal crude solution, and then do the isolation and purification step afterward. This is a gentle way to tune the bandgap of the NCs without destructing the shape and crystal structure of the initial NCs while minimizing the influence of the halide ions on the optical properties. Therefore, while we cannot state that the $PbBr_2$ has no impact on the PLQE whatsoever, but we can affirm that its impact is significantly smaller than that of the $PbBr_2$ post-synthetic surface treatment that is often used for a severely harmed, unwashed NC surface with sufficient surface trap states. Also, the restricted solubility of the ionic salt into coordinating solvents and ligands present in the crude solution at room temperature (after quenching the reaction) would regulate the amount of $PbBr_2$ salt added to the crude solution.

Structural characterisation

Transmission electron microscopy (TEM) measurements were performed using a FEI Tecnai G2 F20 X-TWIN Transmission Electron Microscope with a 200 kV operating voltage. TEM samples were prepared by dropping a dilute colloidal solution of NCs in hexane onto the carbon coated copper grids and dried under ambient conditions.

Compositional characterisation

The concentrations of lead, nickel and zinc ions were determined by using a ThermoFisher Scientific X-SERIES II Quadrupole inductively-coupled plasma mass spectrometer (ICP-MS). For ICP-MS analysis, nanocrystals were stirred overnight in nitric acid to ensure the complete dissolution of the metals into the acid. The measurements were repeated multiple times per composition to confirm a reliable doping concentration in each case.

Steady-state absorption

A Shimadzu UV-3600 Plus spectrophotometer was used to collect the steady-state absorbance spectra of samples, which uses a photomultiplier tube. The final data shown is corrected for by measuring the same cuvette with the solvent (hexane) only and subtracting this spectrum from the one with nanocrystals.

Steady-state and time-resolved photoluminescence (PL)

Steady-state and time-resolved PL spectra were recorded by a gated intensified CCD camera (Andor Star DH740 CCI-010) connected to a grating spectrometer (Andor SR303i). The pulsed output from a mode-locked Ti:sapphire optical amplifier (Spectra-Physics Solstice, 1.55 eV photon energy, 80 fs pulse width, 1 kHz repetition rate) was used to produce 400 nm excitation via second harmonic generation in a β-barium borate crystal. The iCCD gate (width 2 ns) was electronically stepped in 2 ns increments, relative to the pump pulse, to enable ns-temporal resolution of the PL decay. Faster (~100 ps resolved) kinetics were recorded using time-correlated single-photon counting (TCSPC) employing a Picoquant system at 405 nm excitation.

Photoluminescence quantum efficiency (PLQE)

PLQE data was collected using the method described by de Mello *et al.*[1]. Briefly, samples were positioned in an integrating sphere and excited at 400 nm, while the PL was collected with an Andor Shamrock spectrometer and Andor iDus CCD array. A corrected value is then determined by collecting the light from the sphere without a sample, without hitting the sample and with hitting the sample, respectively. Stated values were determined on triplicate samples which were each measured thrice, hence reporting the average of nine measurements for each composition.

Transient absorption (TA) spectroscopy

TA is a form of pump-probe spectroscopy which measures the spectrally resolved variation in absorption by a sample under photoexcitation by a pump source. By varying the pump-probe time delay, the carrier recombination kinetics of the sample can be investigated. The third harmonic of a pulsed Nd:YVO_4 laser (Picolo-AOT MoPa) was used as the pump beam (~1 ns pulse width, 500 Hz repetition rate, 355 nm) for the ns regime measurements. The probe spectrum was generated using a white light quasi-continuum generated through pumping a CaF_2 window with the 800 nm fundamental of a Ti:Sapphire amplifier (Spectra-Physics

Solstice). A delay generator was used to electronically vary the pump-probe delay. For the short time fs-regime, the pump beam was the second harmonic (400 nm) generated by the 800 nm fundamental passing through a β-barium borate crystal. The transmitted probe and reference pulses were recorded with an NMOS linear image sensor (Hamamatsu S8381-1024Q) and processed by a customized PCI interface from Entwicklungsbüro Stresing. For the spin-depolarization measurements, superachromatic quarterwaveplates (Thorlabs) were added to both the pump and probe beam paths, as well as broadband linear polarizers (Thorlabs) before each of these, cleaning up the respective polarization prior to transformation into left- and right-handed circularly polarized light. For co-polarized the same and for counter-polarized the opposite handedness of pump and probe beams was recorded, respectively. In order to not obscure the spectral response of a slightly changing probe beam upon handedness change, the pump polarization was selected to be changed instead. A large pump beam spot size (~1000 µm effective beam diameter) compared to a small probe beam spot size (~300 µm) ensured a high signal stability and homogeneity. Polarization degrees >90% were confirmed for pump and probe prior to each measurement using an analyzing broadband linear polarizer (Thorlabs).

Calculation of excitation recombination rates

We first determine first the total excitation decay lifetime from time-resolved PL. It is found that the PL kinetics for all compositions can only be fitted to a high satisfying level if not less than the sum of three exponentials is used according to:

$$PL(t) = \sum_{i=1}^{n} a_i e^{-t/\tau_X^i} \quad \text{(S1)}$$

with τ_X^i as effective single-exciton lifetime (including both radiative and nonradiative contributions) and a_i the relative fraction (in sum being 1) of NCs in the i-th out of n sub-ensembles possessing this lifetime. The necessity of a triexponential fit is in line with other reports on similar perovskite nanocrystals, for example by the groups of Klimov[2] or Herz[3]. As described in the references, it is assumed that all nanocrystals measured in the ensemble possess the same radiative recombination constant $k_{r,X}$, which is the inverse of the intrinsic radiative lifetime$\langle\tau_{r,X}\rangle$, while the nonradiative recombination constant $k_{nr,X}$ can vary for sub-ensembles, *e.g.* due to different trap densities or identities. From weighting the individual sub-ensemble lifetimes via a_i, we can thus determine the average PL lifetime $\langle\tau_X\rangle$ and hence the average total recombination constant $\langle k_X\rangle = \langle\tau_X\rangle^{-1}$. The PLQE of each sub-ensemble is the ratio of its radiative to total recombination rate, and the total PLQE of the ensemble is therefore:

$$PLQE = \frac{\langle\tau_X\rangle}{\langle\tau_r\rangle} \quad \text{(S2)}$$

From measuring PLQE and total PL lifetimes, the intrinsic radiative lifetime, and hence radiative recombination constant, can be determined. Furthermore, the average non-radiative recombination rate can be calculated, since

$$k_{nr,X} = \langle k_X\rangle - k_{r,X} \quad \text{(S3)}$$

The measured composition-dependent values and extracted recombination constants are summarized in Supplementary Tables T3 and T4.

Calculation of average excitations per nanocrystal $\langle N \rangle$

The excitation fluence per pulse F_{ex} (in µJ cm^{-2}) is related to the laser power P used for photoexcitation of the samples in solution, the repetition rate R_{rep} and the beam spot radius r_{ex} through:

$$F_{ex} = \frac{P}{R_{rep}\pi r_{ex}^2} \quad \text{(S4)}$$

The photon fluence per pulse j (in cm^{-2}) is given by:

$$j = \frac{P\lambda\pi r_{ex}^2}{R_{rep}hc} \quad \text{(S5)}$$

where λ is the excitation wavelength, c is the speed of light in a vacuum, and h is Planck's constant.

The absorbed photon density per pulse j_{abs} (in cm^{-3}) in the cuvette of pathlength l that is being absorbed can be inferred from measuring the UVvis absorbance A at the excitation wavelength and reads:

$$j_{abs} = j\frac{A}{l} \quad \text{(S6)}$$

The density of NCs in solution ρ_{NC} (in cm^{-3}) is related to the NC concentration c_{NC} (in mg mL^{-1}) and the volume of a NC V_{NC} and the weight density of the material ρ_m via:

$$\rho_{NC} = \frac{c_{NC}}{V_{NC}\rho_{NC}} \quad \text{(S7)}$$

Therefore, the average excitations per NC $\langle N \rangle$ determined by this approach is:

$$\langle N \rangle = j_{abs}/\rho_{NC} \quad \text{(S8)}$$

Calculation of wavefunction overlap of electrons and holes

The wavefunction overlap Θ of electrons and holes is defined as:

$$\Theta = |\int \psi_e^* (r)\psi_h(r)dV|^2 \quad \text{(S9)}$$

Following an approach previously used for example by the groups of de Mello Donegá[4,5], or Kelley[6,7] and originally described by Efros and Rodina[8], the overlap integral is directly related to the oscillator strength and radiative rate via:

$$k_{r,X} = \frac{2e^4n}{\pi\varepsilon_0 m_0^3 c^3}|F|^2\frac{m_0^2 E_g E_p \Theta}{3e^2\hbar^2} \quad \text{(S10)}$$

where e is the elementary charge, n the refractive index, ε_0 is the vacuum permittivity, c is the speed of light in a vacuum, m_0 is the free electron rest mass, $\hbar$ is the reduced Planck's constant, and E_g is the energy of the optical transition.

$F = 3\varepsilon_m/(\varepsilon_s + 2\varepsilon_m)$ is the local field factor to account for the screening of the nanoparticle, assuming a random orientation of NCs with respect to the electric field of the interacting light. For simplicity, we modelled the NCs as spheres, with ε_m and ε_s being the dielectric constants of the medium and semiconductor, respectively. A more rigorous treatment of the electric field strength inside cubic nanocrystals can be found in ref.[9] Importantly, this will not influence the changes to the wavefunction overlap, since the same shape of NCs is maintained without and with doping, as checked with TEM. E_p is the Kane energy, usually found for III-V semiconductors to be on the order of 20 eV[2], but notably very close to 40 eV for $CsPbX_3$ perovskites due to the different orbital contributions to the valence and conduction band edges here, independent of halide choice[9]. The formal derivation of Eq. S10 can be found in the early works by Efros[8,10], and later also discussed together with Bawendi[11].

Importantly, the measured increases in oscillator strength (here extracted from time-resolved PL & PLQE; for the inverse approach via transient absorption, see our detailed study on the manganese system where we show the equivalence of both measurements explicitly[12]) and thus also in the radiative rate *cannot* be explained by the only slight increase in bandgap and Kane energy upon doping. This is because the doping level in the percent-regime is too small to significantly alter the global properties of the semiconductor, while still an exciton will experience the presence of a dopant, given that the distance between the distributed ions is less than 3 nm and the exciton Bohr radius already approx. 2.5 nm (then followed by diffusion as well). Thus, the overlap integral increases significantly in order to account for the enhancement observed experimentally. This stronger overlap is a direct consequence of the lattice-periodicity breaking by the dopants which leads to the localization of charges, thereby increasing the radiative recombination rate of charges in their vicinity.

First principles calculations

Computational methods and parameters:

Density functional theory (DFT) calculations were performed using the Vienna ab initio simulation package (VASP)[13,14]. The core-valence interaction was described using the projector-augmented wave (PAW) method[15,16], with 9 valence electrons for Cs ($5s^2 5p^6 6s^1$), 14 valence electrons for Pb ($5d^{10} 6s^2 6p^2$), 7 valence electrons for Cl ($3s^2 3p^5$) and Br ($4s^2 4p^5$), 13 valence electrons for Mn ($3p^6 3d^5 4s^2$), 10 valence electrons for Ni ($3d^8 4s^2$), and 12 valence electrons for Zn ($3d^{10} 4s^2$). Due to the presence of the heavy Pb atom, all electronic structure calculations except structural relaxation were done including spin-orbit coupling (SOC) effects, which are included perturbatively to the scalar-relativistic Hamiltonian[17].

We first performed geometry optimizations of the undoped cubic $CsPbBr_3$ and $CsPbCl_3$ where the atomic positions and lattice parameters were allowed to fully relax using the semi-local PBEsol functional[18]. The electronic wavefunctions were expanded in a planewave basis with an energy cut-off of 400 eV, the Brillouin-zone was sampled with a 6×6×6 Γ-centered Monkhorst-Pack[19] k-point grid and the Hellmann-Feynman force convergence threshold was 0.01 eV/Å.

For the doped structures we constructed a 3×3×3 supercell from the relaxed cubic unit cell of $CsPbBr_3$ and $CsPbCl_3$, and a single Pb atom was replaced with Ni/Zn atom, for a doping concentration of 3.7%. The supercells were then relaxed with the lattice parameters fixed, and only the atomic positions allowed to move. At this doping concentration, we found fully relaxing both lattice parameters and atomic positions results in negligible structural changes. For the supercell calculations we employed a commensurate k-point grid of 2×2×2. For Ni doping, a ferromagnetic spin configuration was assumed, and for Zn doping, a non-spin-polarized

calculation was used. To obtain the band structure, we used the PBEsol functional with SOC effects. The effective masses were directly calculated from the band structure *via* a parabolic fit (R → Γ) using the formula $\frac{1}{m^*} = \frac{1}{\hbar^2}\frac{\partial^2 E}{\partial k^2}$. It should be noted that the curvature of the band strongly depends on the functional choice and that our results should only be taken as a qualitative measure, but do match the observed experimental relative trends very well.

For the projected density of states (PDOS) calculation, we used the PBE0 functional[20,21]. To reduce the computation load, a lower energy cut-off of 300 eV was used, which can still reproduce the electronic structure accurately[22]. The bandgap was extracted at the R point. Mn was independently studied in a previous work we have published in ref.[12] using the HSE06 functional that calculates a slightly lower bandgap for the pristine and the doped system, the exact computational parameters can be found in the SI therein. We employed a scissor shift to the PDOS such that the optical bandgap matches the results using the PBE0 functional for better comparison.

Influence of halide mixing on the symmetry analysis:

We note that the mixing of halides alone in principle also breaks the translational symmetry of the pure halide species. However, perovskites are highly miscible on the halide sites such that many electronic (*e.g.* band gap, zero bowing parameter) and structural properties (lattice parameter) follow a linear trend as a function of the halide composition[23,24]. This high tolerance for halide alloying allows us to treat the mixed halides as a single atomic species with extrapolated electronic properties between that of chloride and bromide. This has been validated using the so-called “virtual crystal approximation”, which effectively creates a uniform potential background with the symmetry of the pure halide species[25,26].

Supplementary Data

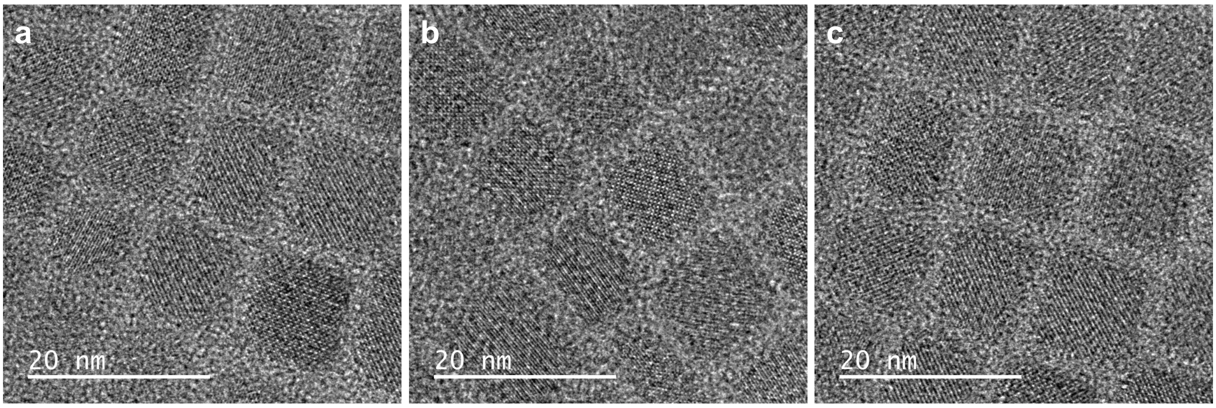

Supporting Figure S1: Scanning transmission electron microscopy images of pristine (a), nickel (b) and zinc (c) doped perovskite nanocrystals (NCs), respectively. See ref.[12] for manganese case. All undoped and doped NCs show a cubic morphology and an average crystal size of 10±2 nm, irrespective of composition, placing them in the weak quantum confinement regime. Scale bar is 20 nm, respectively.

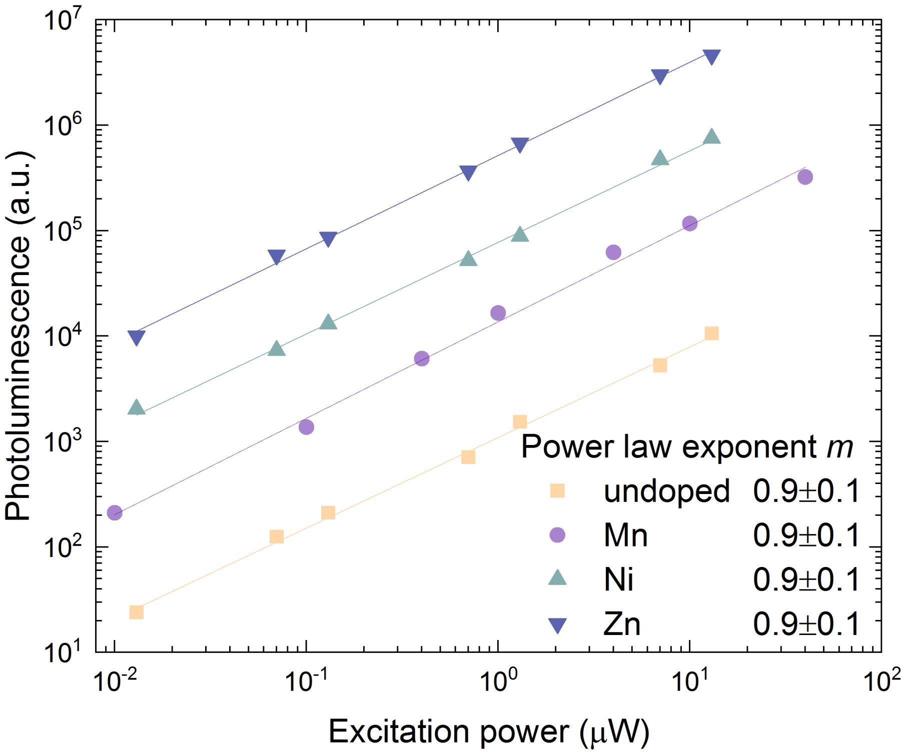

Supporting Figure S2: Excitation power dependence of photoluminescence intensity. Data traces are vertically off-set for clarity. The fit according to P^m confirms a linear PL dependence on excitation power (P), expected for excitonic recombination.

$PbBr_2$ (g)	$PbCl_2$ (g)	$NiCl_2$ (g)	$PbBr_2$ (mmol)	$PbCl_2$ (mmol)	$NiCl_2$ (mmol)	Molar ratio Ni/Pb	ICP-MS Ni^{2+} (%)
0.165	0.0836	0	0.450	0.301	0	0	0
0.21	0	0.071	0.572	0	0.547	0.956	1.10%
0.21	0	0.081	0.572	0	0.625	1.092	2.01%

$PbBr_2$ (g)	$PbCl_2$ (g)	$ZnCl_2$ (g)	$PbBr_2$ (mmol)	$PbCl_2$ (mmol)	$ZnCl_2$ (mmol)	Molar ratio Zn/Pb	ICP-MS Zn^{2+} (%)
0.165	0.0836	0	0.450	0.301	0	0	0
0.21	0	0.071	0.572	0	0.520	0.909	1.50%
0.21	0	0.081	0.572	0	0.594	1.038	1.55%

Supporting Table T1: Typical amounts of precursors used for the Ni^{2+} and Zn^{2+} doped nanocrystals and atomic doping levels measured in the final NC samples. For a detailed doping concentration dependence study on Mn^{2+} see ref.[12].

Composition	Urbach energy E_U of absorbance (eV)	*FWHM* of PL (eV)
undoped	0.018±0.005	0.113±0.005
Mn	0.017±0.005	0.113±0.005
Ni	0.021±0.005	0.111±0.005
Zn	0.019±0.005	0.112±0.005

Supporting Table T2: Energetic disorder as a function of doping. An Urbach-energy-like expression for the energetic disorder in the absorbance $A = A_0 e^{\left(\frac{E-E_c}{E_U}\right)}$, with A and E_c being fitting parameters, and E_U a measure for the energetic disorder, as well as a Gaussian fit to the PL spectra for extracting the full-width at half-maximum (FWHM) for the emission has been used to fit the data in Figure 1 of the main text, but did not show any significant energetic broadening upon doping.

Composition	$\tau_1\ (ns)$	$\tau_2\ (ns)$	$\tau_3\ (ns)$	A_1	A_2	A_3
undoped	1.334	3.443	10.815	38142	38985	1839
Ni low	1.504	3.762	11.143	39862	24972	2479
Ni high	1.470	3.529	10.081	47765	18888	1859
Zn low	1.325	3.594	10.573	30013	24893	2675
Zn high	1.383	3.563	10.676	4439	1727	1480

Supporting Table T3: Fit parameters extracted from a triexponential fit to the data shown in Figure S3 below.

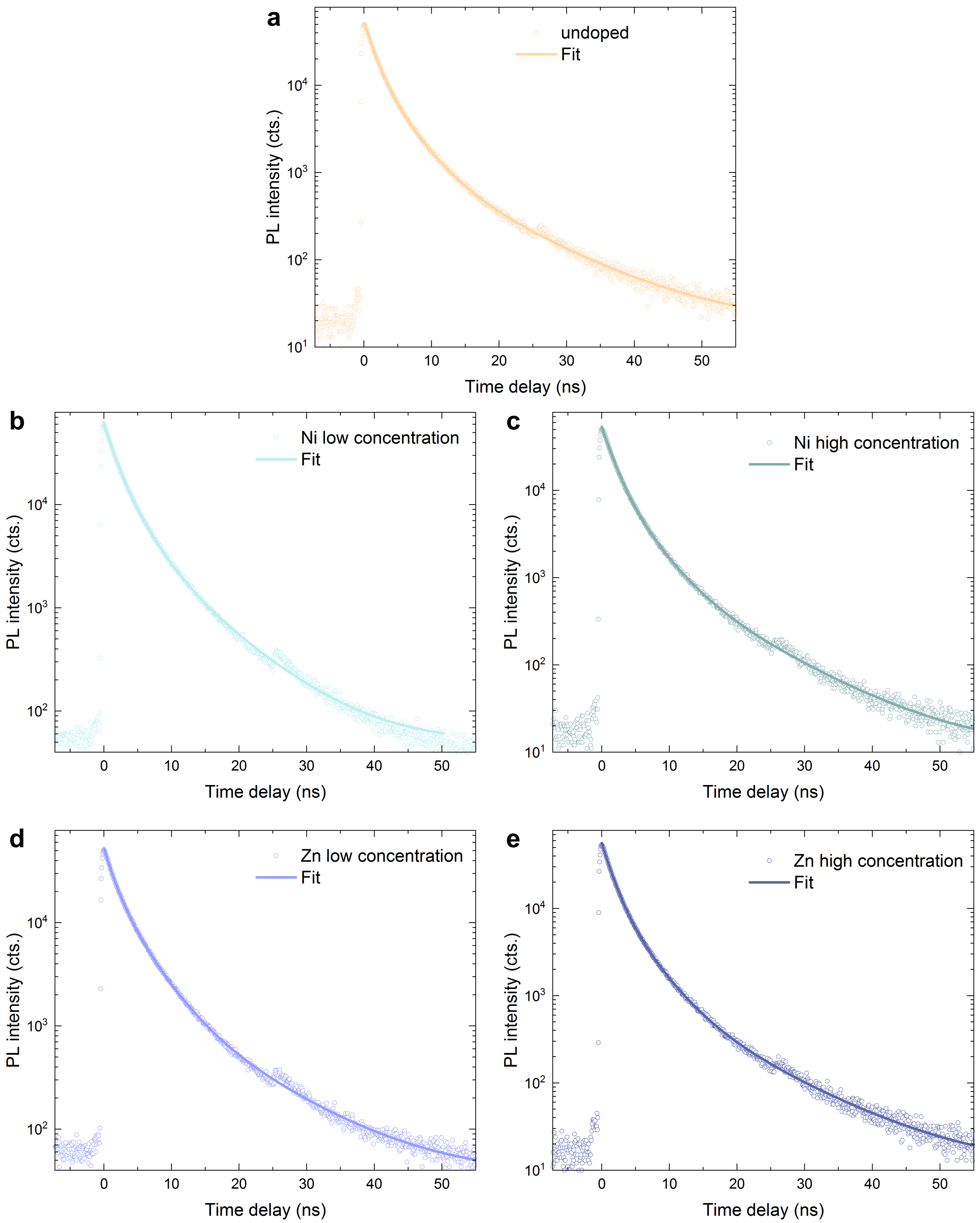

Supporting Figure S3: Time-resolved photoluminescence decays for **a**, undoped, **b**, 1% Ni doped, **c**, 2% Ni doped, **d**, 1% Zn doped, and **e**, 2% Zn doped nanocrystal dispersions in hexane. All samples excited with low-fluence 405 nm pulses (length ~500 ps) using the time-correlated single-photon counting method and integrated over the full spectrum. Bold lines show triexponential fits used for average lifetime determination for each composition (results summarized in table below). See ref.[12] for the full data set on Mn we reported earlier. The systematic small feature at 25 ns in all measurements is an instrument artefact due to pump

scatter, as confirmed by measurements on a dispersion of scattering titania particles showing the same response, and did not influence the fit parameters extracted.

Composition	$\langle k_X \rangle$ (10^8 s^{-1})[a]	PLQE (%)[a]	$k_{r,X}$ (10^8 s^{-1})	$k_{nr,X}$ (10^8 s^{-1})
undoped	3.85	34	1.29	2.55
Ni low	3.71	41	1.51	2.20
Ni high	4.40	48	2.11	2.29
Zn low	3.66	44	1.63	2.02
Zn high	4.55	53	2.37	2.18

[a]Measurements taken at 405 nm excitation at a low-energy flux of 109 μW cm^{-2}.

Supplementary Table T4. Atomic doping simultaneously enhances radiative and decreases non-radiative recombination. The inverse of the average PL lifetime, as determined from TCPSC (Fig. S2), $\langle k_X \rangle$, and the non-radiative recombination constant, $k_{nr,X}$, are reduced upon doping, while the PLQE and the radiative recombination constant, $k_{r,X}$, increase upon doping, with zinc showing stronger improvements than nickel under otherwise identical conditions. $k_{r,X}$ was calculated according to $k_{r,X} = \mathrm{PLQE} \times \langle k_X \rangle$ and $k_{nr,X}$ was calculated according to $k_{nr,X} = \langle k_X \rangle - k_{r,X}$, as discussed earlier, *e.g.* by Klimov and co-workers.[2] Similar results for manganese doping can be found in our earlier study focused on this dopant[12] and the linear increase in radiative rate with doping experimentally confirmed there was used for scaling the value shown in Fig. 1 to reflect the same doping level as for the other dopants.

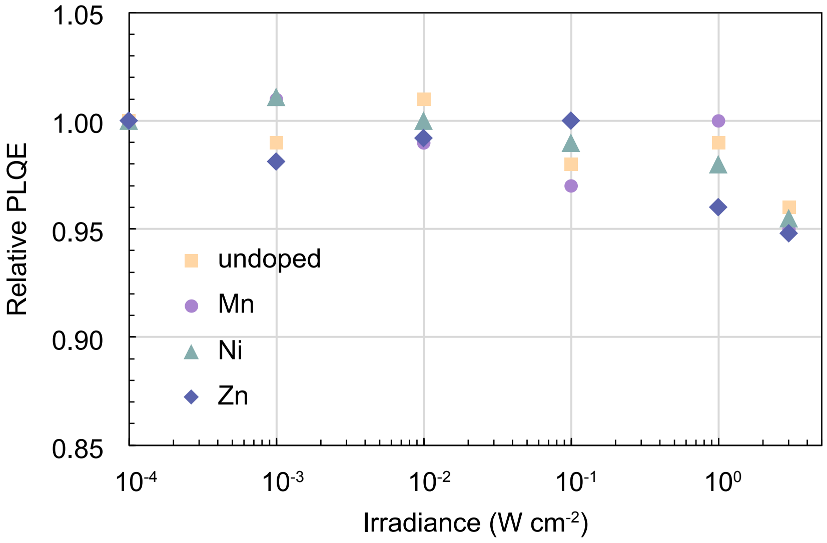

Supporting Figure S4: Relative PLQE calculated as integrated PL intensity divided by Irradiance. Within the sensitivity of the measurement, a constant relative luminescence yield is observed, supporting an excitonic recombination mechanism throughout the majority of excitation fluences studied. At the highest fluence a reduction in luminescence yield suggests the onset of exciton-exciton annihilation.

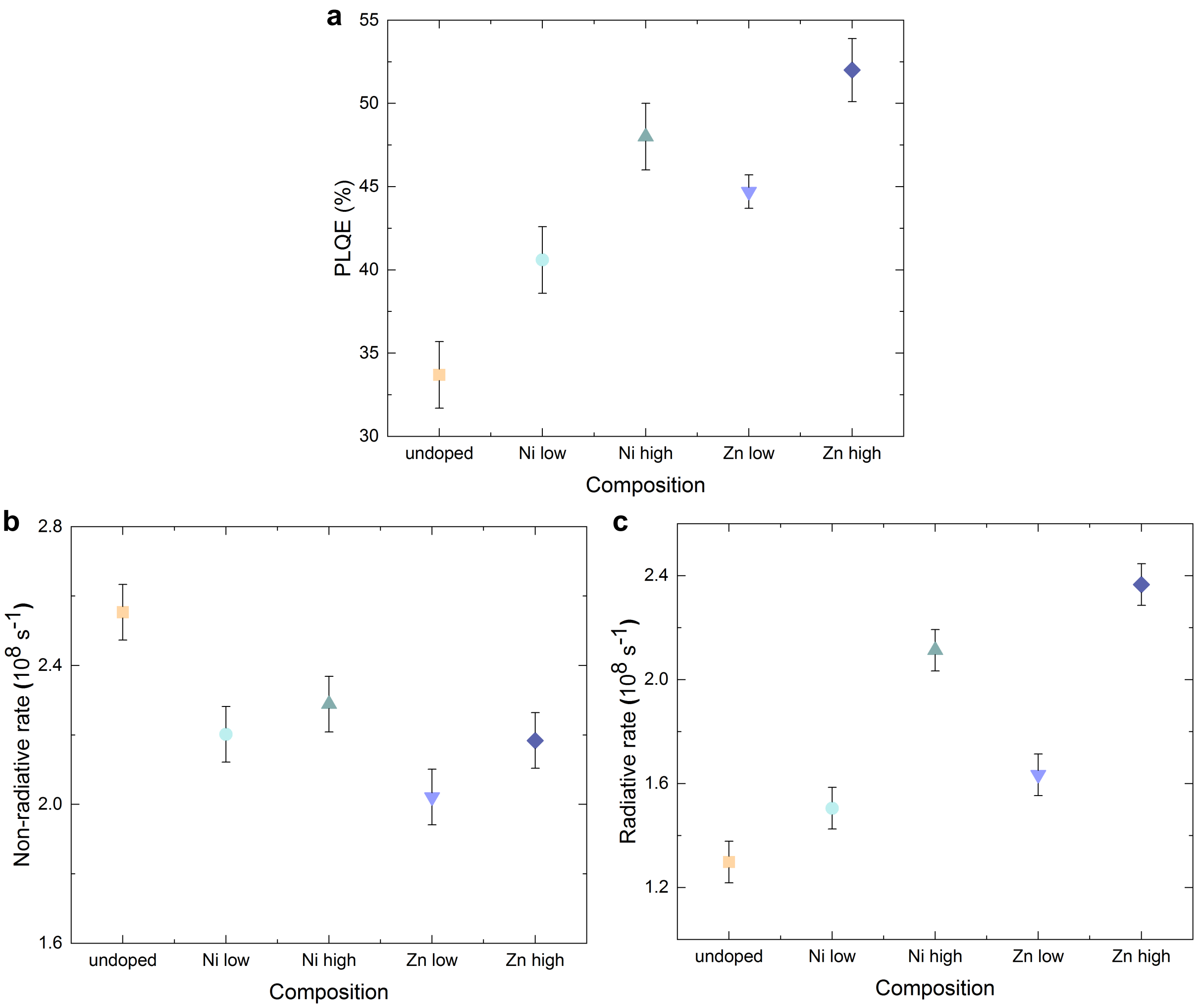

Supporting Figure S5: Concentration dependence of doping-induced changes to the NC emission properties. **a**, External PLQE, increasing upon doping with increasing doping concentration for Ni and Zn doping, respectively. **b**, Non-radiative recombination rate, which is reduced upon doping, and most efficiently so at low doping concentration, respectively. **c**, Radiative recombination rate, which increases upon doping, and increases with doping concentration. All samples were photoexcited at 405 nm at a low-energy flux of 109 μW cm^{-2} (pulse length ~500 ps).

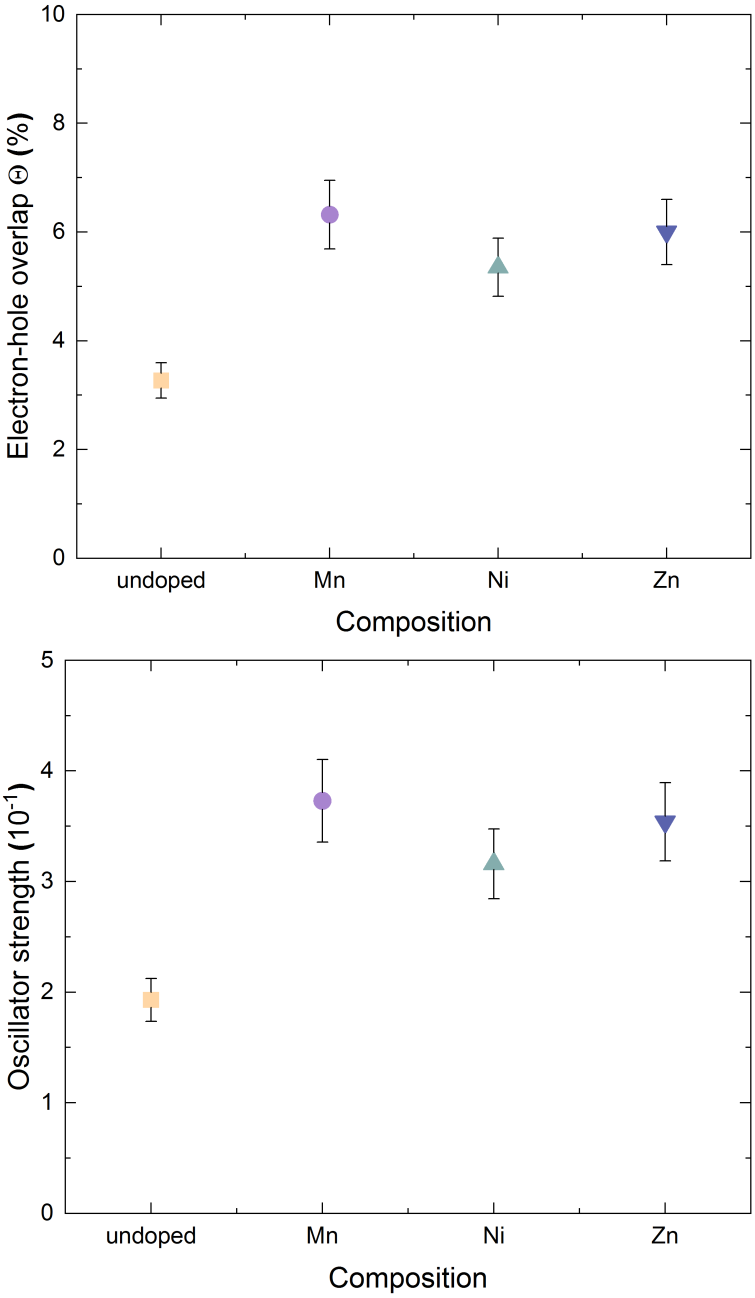

Supporting Figure S6: Overlap integral (top) and oscillator strength (bottom) extracted from experimental data (see Methods section for details). Both observables increase significantly upon doping due to lattice periodicity breaking.

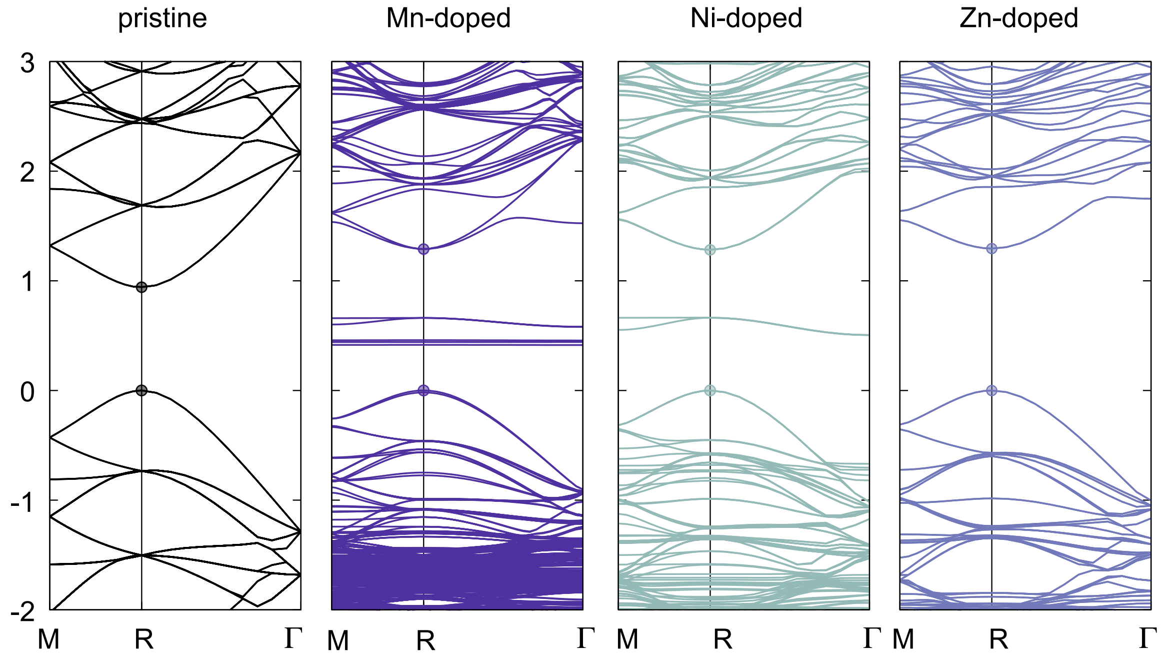

Supporting Figure S7: $CsPbCl_3$ band structures of the pristine and transition-metal doped perovskite (nominal doping concentration of 3.7%) calculated using the PBEsol functional with SOC. The effective masses were extracted using the perovskite band edges highlighted with circles. Black: band structure of pristine 3×3×3 $CsPbCl_3$ supercell with a direct band gap at the R-point. Purple: band structure of Mn-doped $CsPbCl_3$ with in-gap states corresponding to Mn d-states. We highlight that the position of the d-states is lifted towards the perovskite band edges when more accurate hybrid functionals are used in the main manuscript. Green: band structure of Ni-doped $CsPbCl_3$, again showing in-gap bands corresponding to dopant d-states. These are pushed into the conduction bands when PBE0 is used. Blue: band structure of Zn-doped $CsPbCl_3$.

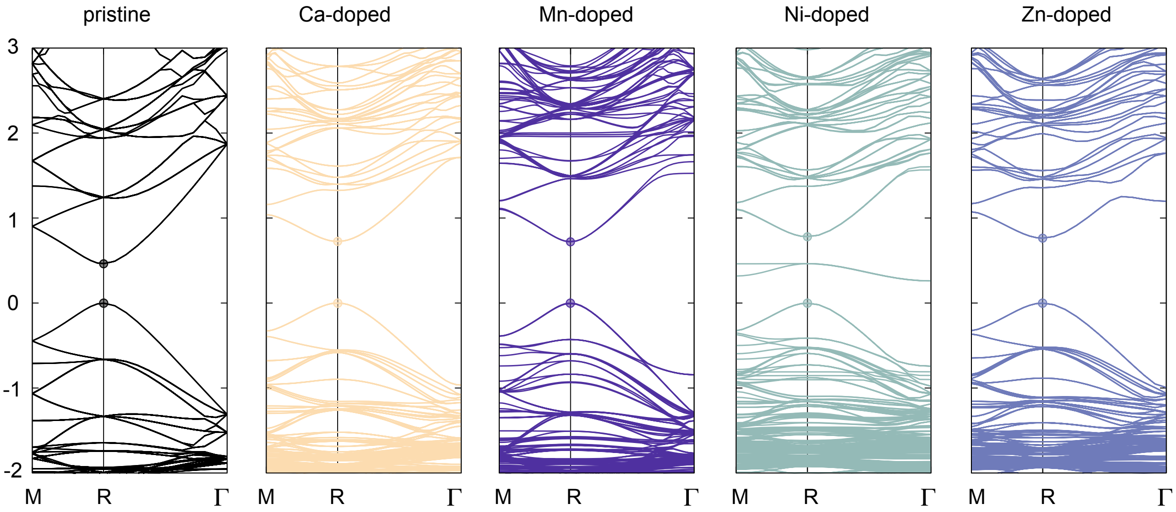

Supporting Figure S8: $CsPbBr_3$ band structures of the pristine and transition-metal doped perovskite (nominal doping concentration of 3.7%) calculated using the PBEsol functional with SOC. The circles highlight the band edges that were used to calculate the effective masses.

Black: band structure of pristine 3×3×3 $CsPbBr_3$ supercell with a direct band gap at the R-point. Yellow: band structure of Ca-doped $CsPbBr_3$. The Ca-doped effective masses were extracted as 0.144 (hole) and 0.15 (electron) electron masses, respectively. Purple: band structure of Mn-doped $CsPbBr_3$. Green: band structure of Ni-doped $CsPbBr_3$, again showing in-gap bands corresponding to dopant d-states. These are pushed into the conduction bands when PBE0 is used. Blue: band structure of Zn-doped $CsPbBr_3$.

<u>Study on alkaline-earth metal doping on $CsPbBr_3$</u>

To further study the role of ionic radii, concentration, and element-specific changes we conducted a thorough complementary first-principles study on the B-site doping of $CsPbBr_3$ with the alkaline earth (AE) metals beryllium (Be), magnesium (Mg), calcium (Ca), strontium (Sr) and barium (Ba). We have chosen the AE elements because they capture a wide range of ionic radii, electronegativities, electron affinities and all have unambiguous oxidation states of +2 that conserves the total charge of the crystal. Furthermore, due to the electron configuration of ns^0 we assume that both the filled and the empty dopant orbitals are well isolated from the perovskite band edges.

Again, all calculations are performed using density functional theory (DFT) with the projector augmented wave method as it is implemented in the Vienna Ab Initio Simulation Package. For the geometry optimisations we employed an energy cut-off 350 eV and a Γ-centred Brillouin zone (BZ) grid including 6×6×6 *k*-points for the primitive cell and commensurate grids for the supercells to relax the volume until the stress is below 10^{-2} GPa, while the internal atomic coordinates are fixed by symmetry. We perform all geometry optimisations using the generalised-gradient approximation of Perdew, Burke, and Ernzerhof (PBE) without the inclusion of spin-orbit coupling. We subsequently calculate the electronic properties using the same parameters but now we include the effect of spin-orbit coupling using the second variational method.

We start from a pristine primitive cell describing cubic $CsPbBr_3$. As an initial structural guess, we use the primitive cell as above and construct 2×2×2, 3×3×3 and 4×4×4 supercells. These supercell sizes give a sufficiently good range of doping concentrations for deducing trends but without reaching computationally prohibitive limits. We then introduce dopants into the pristine supercells through B-site substitution of one Pb atom. With the aforementioned range of supercell sizes, we are able to reach doping concentrations of up to 1.56%. In the first step we calculate the band gap of the doped systems without allowing the geometry to relax after substitution. This set of calculations allows us to deduce the effect of particle substitution of each AE atom on the band gap without any structural distortions arising from an ionic radius mismatch.

To understand how structural adaptation to the dopant changes the band gap, we then allow the internal coordinates to relax without changing the volume of the simulation cell. The atoms within the cell are allowed to move and twist to change the bond lengths and angles to accommodate for the new dopant.

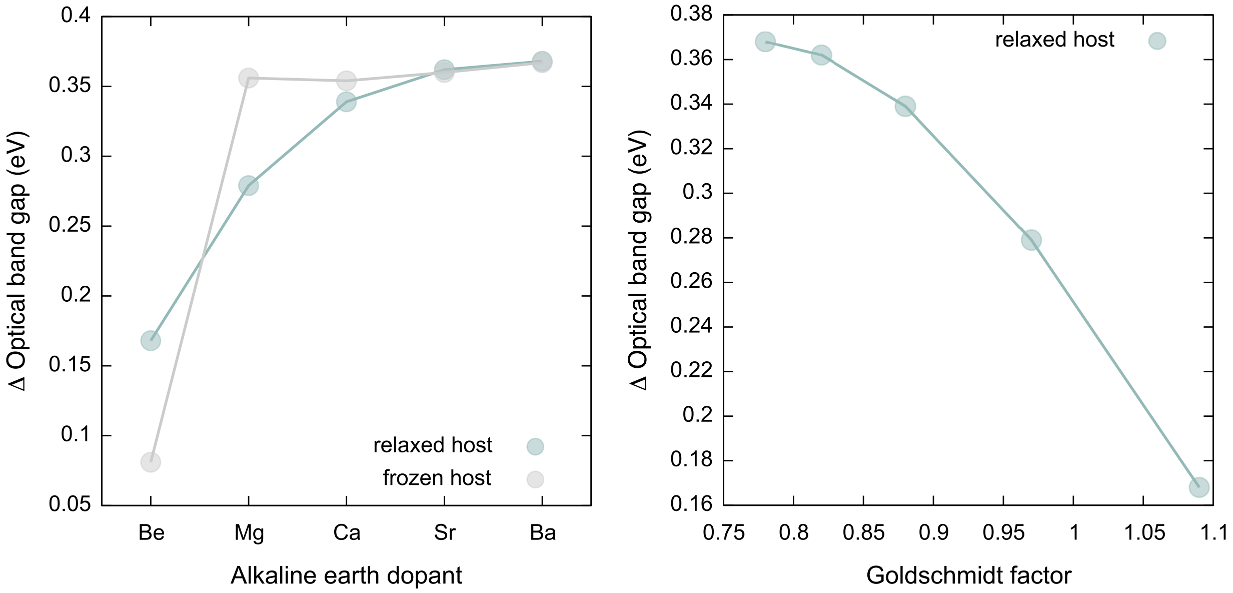

Supporting Figure S9: Dopant-dependent band-gap changes at a nominal doping concentration of 12.5%**.** Left**,** Calculated bandgap change in $CsPbBr_3$ upon doping with different alkaline earth (AE) metals. Grey: In this scenario, the replacement of a Pb atom with an AE atom is not followed by a structural relaxation. Green: In this scenario, the replacement of Pb with an AE atom is followed by a structural relaxation. At high concentrations, the structural adaptation of the perovskite scaffold leads to a blueshift with respect to the frozen configuration (with Be being the exception because it exhibits an in-gap state). Right, Change of the optical band gap against the Goldschmidt tolerance factor. The almost identical slope of the curve indicates that the size mismatch is the dominating factor that governs the difference between the frozen and the relaxed scenario.

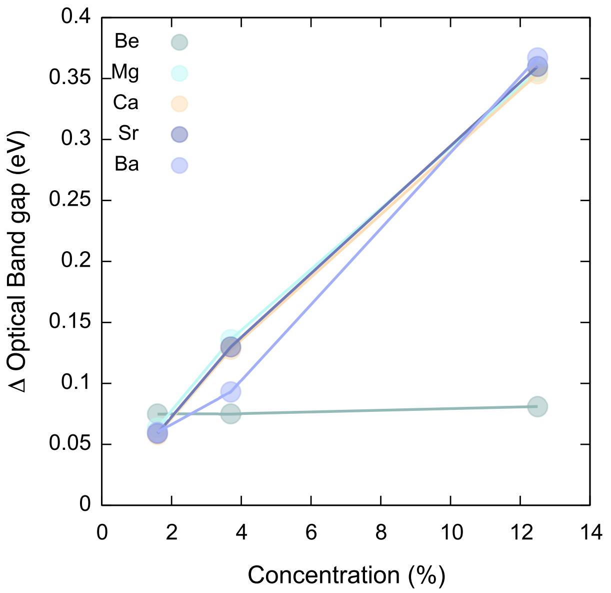

Supporting Figure S10: Calculated change of the band gap of $CsPbBr_3$ as a function of nominal doping concentration of all the alkaline earth dopants. In this case, the host structure is kept frozen. The concentration-dependent band-gap opening is linearly dependent on the

nominal doping concentration (with Be being the exception because it exhibits an in-gap state), which is a result of breaking the periodicity of the Bloch states and pushing them towards the atomic limit.

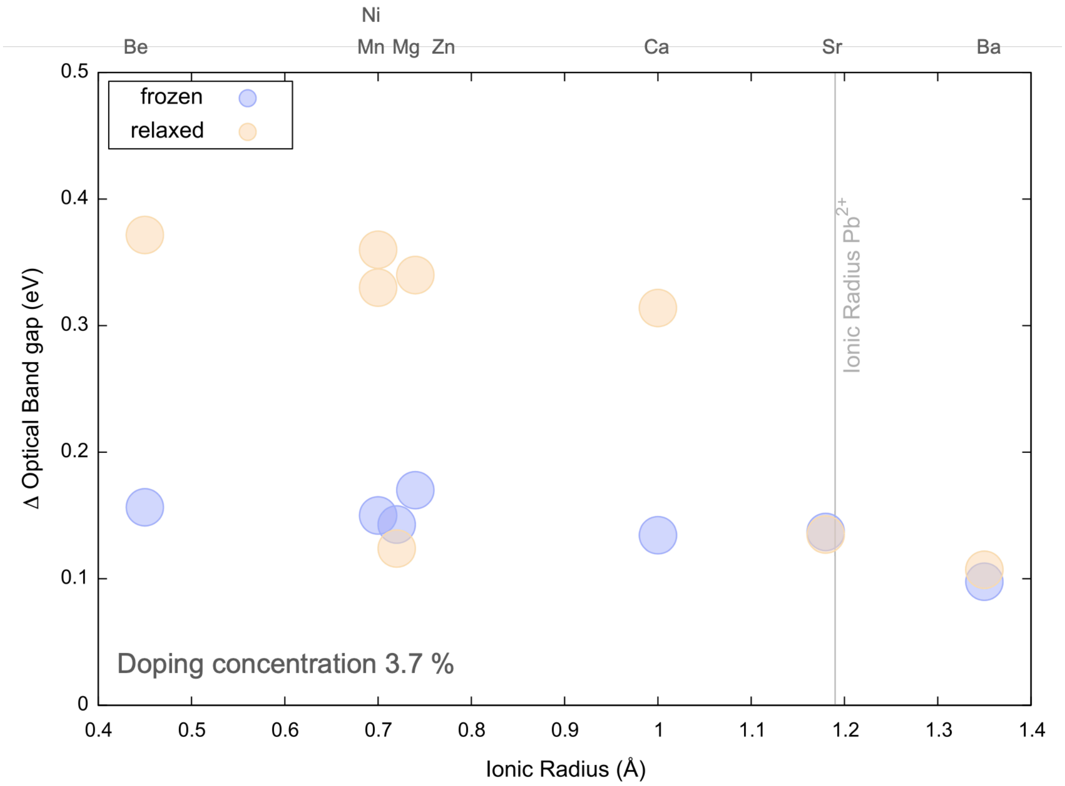

Supporting Figure S11: Change of the band gap as a function of the dopant ionic radius (Å) at low concentrations. Here, the optical band gap was calculated for the frozen as well as for the relaxed perovskite scaffold. It becomes evident that the structural relaxation of the host as a response to the B-site doping with a size-mismatched dopant (we added the transition metal results for completeness here) leads to a further band gap opening. This can be understood in terms of structural distortions that are captured in a bigger supercell but cannot be captured in small supercells. Here we see octahedral tilts in the perovskite scaffolds that are not present at higher concentrations (*i.e.* smaller supercells). Octahedral tilts in lead halide perovskites are known to widen the optical band gap due to the perturbed orbital interaction between the Pb and the halide atoms. This effect diminishes at strontium which has the same ionic radius as Pb and thus does not cause any structural changes due to ionic size mismatch.

Mechanistic origin of the band gap opening and effective mass increase

As highlighted in the main text, the response of the optoelectronic properties upon B-site doping (at low concentrations) follows very clear trends which we explain in a chemically intuitive fashion, bridging the gap between molecular orbital (MO) and electronic structure theory, using the linear combination of atomic orbitals (LCAO) ansatz and Bloch's theorem. For our discussion we closely follow the arguments of Goesten and Hoffmann. For the sake of simplicity, we do not include spin-orbit effects in this model as the symmetry arguments proposed here can also be used when spin-orbit effects are included.

We start with the smallest building block of the cubic perovskite structure as it is illustrated in Figure S11a. Here, the PbX_3 unit (Cs is omitted as it does not contribute to the relevant electronic states) is used to construct a cubic periodic lattice along the lattice vectors $a_1=a_2=a_3$, which are parallel to the Pb-X bonds of the PbX_3 unit. This leads to the cubic crystal structure. We also use the atomic PbX_3 unit to construct the near-band electronic states in the infinite lattice. For this, we show in Figure S11b the relevant atomic orbitals (AOs) that contribute to the electronic states close to the Fermi level, namely the Pb 4s and 4p, as well as the halide p.

For the PbX_3 unit with C_{3v} symmetry we construct symmetry-adapted linear combinations of the halide p orbitals that create a sigma-type overlap with the Pb 4s orbital, creating a stabilized σ fragment molecular orbital (FMO) with three bonding interactions, as well as a destabilized σ^* FMO with three antibonding interactions. We analogously construct another set of FMOs, now using the Pb 4p and halide p AOs, which we refer to as the $\sigma\pi\pi$ basis. This symmetry-adapted linear combination leads to a threefold degenerate $\sigma\pi\pi$ and $\sigma^*\pi^*\pi^*$ basis. These FMOs do not contain the halide s orbitals but are sufficient for a qualitative description of the valence band (VB) and the conduction band (CB).

Using the FMOs we can build the delocalized Bloch states in the cubic perovskite crystal at the high-symmetry points Γ and R, according to Figure S12a. Key features of cubic lead halide perovskite which makes the electronic structure so chemically tangible are 1) that the lattice vectors are parallel to each Pb-X bond and 2) that the real and the reciprocal lattice vectors are parallel to each other (and hence the1st BZ is cubic as well). Therefore, we can create the Bloch states for the zone center Γ and zone edge R along the real-space lattice vectors, now only including additional phases for different wavevectors k. We show the construction of the Bloch states along a reciprocal lattice direction below. For Γ, no phase change between different FMO units occurs, whereas at the R-point a phase change between each neighbouring FMO occurs. This picture, however, is yet incomplete and does not show us whether the generation of the Bloch states stabilizes or destabilizes the energy at the different high-symmetry points. For this it is necessary to consider the type of orbital interaction that occurs upon imposing translational symmetry. What happens with the different FMOs at Γ and R is depicted in Fig. S12b. For clarity, we only show the truncated PbX_6 octahedron. The σ and σ^* basis at Γ interact such that the octahedron exhibits three bonding and three antibonding interactions, making the states overall non-bonding and stabilizing them (the Bloch state corresponding to the σ^* FMO is stabilized w.r.t. the FMO and the state corresponding to the σ^* FMO is stabilized w.r.t. the FMO). In contrast, at R we see for the σ and the σ^* FMO six bonding and no antibonding, and six antibonding and no bonding interactions, respectively. This then suggests that the σ^* states (which build the valence band) are most stabilized at the Γ point and most destabilized at the R-point (which is where the VBM is located). For the $\sigma\pi\pi$ and $\sigma^*\pi^*\pi^*$ basis the opposite is true and here we will only consider the $\sigma^*\pi^*\pi^*$ basis, since it builds the CB. At Γ, the state exhibits six antibonding

interactions, destabilizing it w.r.t. the FMO, whereas at the R-point it only has three bonding and three antibonding interactions. These interactions result in a stabilization and destabilization of the Bloch state with respect to the FMOs at the Γ-point and the R-point, respectively.

Now that we understand how the translational symmetry stabilizes and destabilizes the electronic states along the Γ→R high-symmetry line, we can easily derive the effect of breaking translational symmetry by incorporating a B-site dopant.

In Fig. S13a we have illustrated the effect of doping on the band edges at R. Since the VB is the most antibonding at R (thus creation of a symmetry adapted linear combination through translational symmetry increases the energy of the Bloch state), breaking the periodicity will reduce the destabilization, pushing the VBM down in energy. The opposite is true for the CBM, where breaking the periodicity will push the CBM up because it perturbs the bonding interaction between the neighbouring FMOs. Both effects combined lead to an increase of the electronic band gap for every B-site dopant, as we have determined both experimentally and from first principles calculations.

This symmetry-encoded response to the dopant also explains the reduction of the effective charge carrier masses. Here, we consider the slope of the electronic bands along the R→Γ high-symmetry line and calculate the effective masses using a parabolic fit of the VB and the CB (which depend on the slope of the electronic band). Starting at the R-point of the VB, the state with the strongest antibonding interactions of the band, we move towards the zone center. Along that path, the strength of the antibonding interaction due to translational symmetry is continuously weakened until it reaches Γ, where the band is the most bonding. Breaking periodicity by incorporating a B-site dopant will cause the strongest stabilization effect at the R-point (since the translational symmetry induced antibonding interaction is the strongest at R), with the effect ceasing along the R→Γ high-symmetry line, up to a point, at which the bonding interaction dominates in proximity to the Γ-point (and thus breaking periodicity destabilizes the electronic states close to the zone center). This effect is illustrated in Fig. S13b. Considering the conduction band, the exact opposite effect occurs: Breaking periodicity causes the CB to destabilize most at the R-point and to progressively destabilize less, when we move along the R→Γ high-symmetry line. The effect on both the valence and conduction band leads to a change in the slope, which we qualitatively quantify using the bandwidth, and consequently to a reduced effective mass of charge carriers (*i.e.* reduced delocalization). We note that full first principles calculations show that the R→Γ high-symmetry line may cross with defect levels, but this should not affect the conclusions drawn here about the bandwidth.

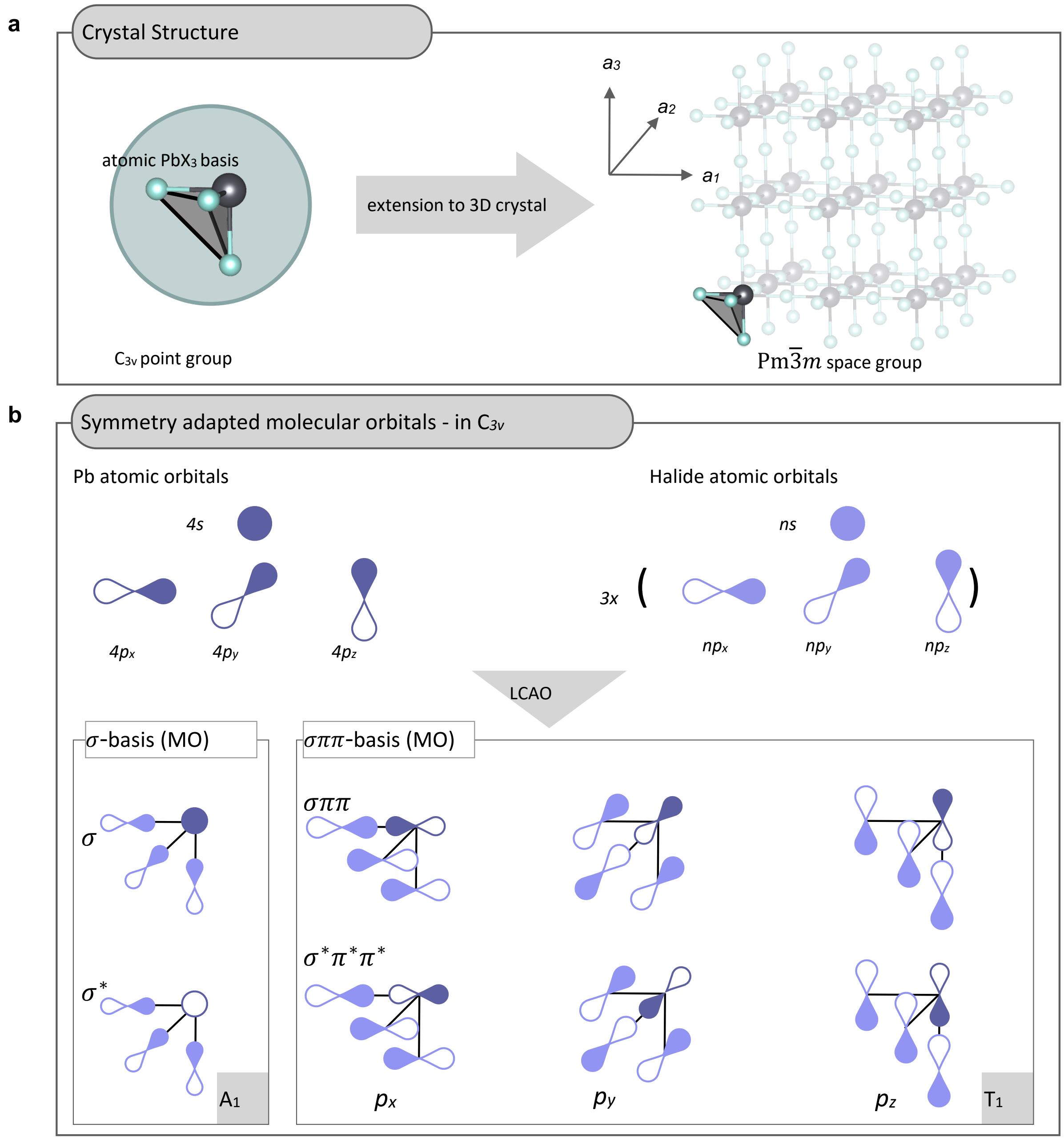

Supporting Figure S12: a, Illustration of the PbX_3 atomic basis with the point group C_{3v} that is used to build the cubic perovskite crystal lattice by translation of the basis along the cubic lattice vectors $|a_1|=|a_2|=|a_3|$. The Cs atom is omitted here since it only negligibly contributes to the formation of the bands close to the band gap. In this crystal, each Pb lies at the Wickoff position *1a* (0,0,0) and has a point group symmetry of O_h. **b,** Top, valence atomic orbitals of lead and halide atoms. Bottom left, linear combination of atomic orbitals (LCAO) generates bonding and antibonding molecular orbitals of the atomic PbX_3 unit with a_1 symmetry (within the C_{3v} point group). Bottom right, bonding and antibonding molecular orbitals of the atomic PbX_3 unit with t_1 symmetry (within the C_{3v} point group).

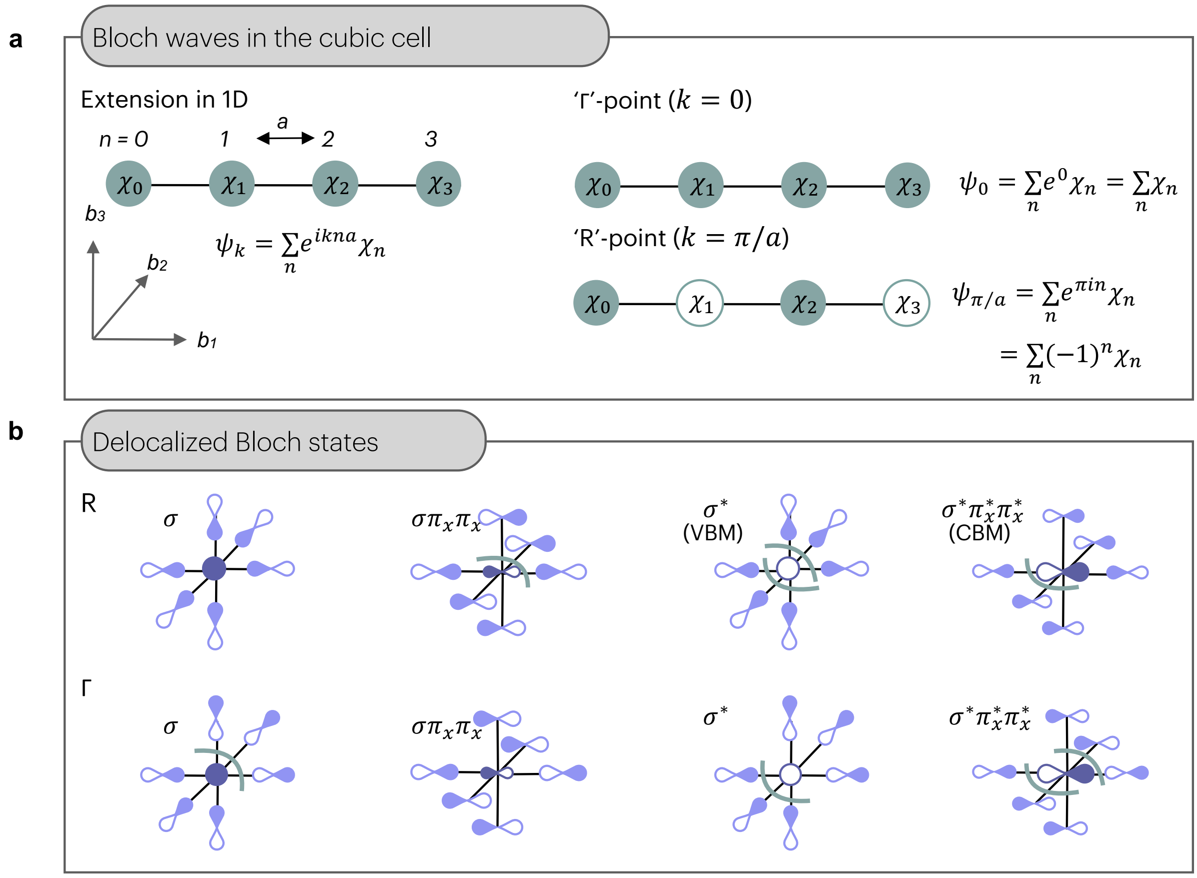

Supporting Figure S13: a, Formation of Bloch states along one of the identical reciprocal lattice vectors *b* from any of the molecular bases of PbX_3 in a cubic lattice. At the Γ-point, there is no phase change between the repeating PbX_3 units, whereas at R the phase changes between each neighbouring PbX_3 unit. **b,** Formed Bloch states at the high-symmetry points Γ and R, truncated in order to illustrate the bonding and antibonding interaction as well as the symmetry of the isolated PbX_6 octahedral MOs. The green lines show the antibonding interactions between the Pb and the halide orbitals. The size of the orbital lobes is a qualitative measure of the energy-dependent orbital coefficients.

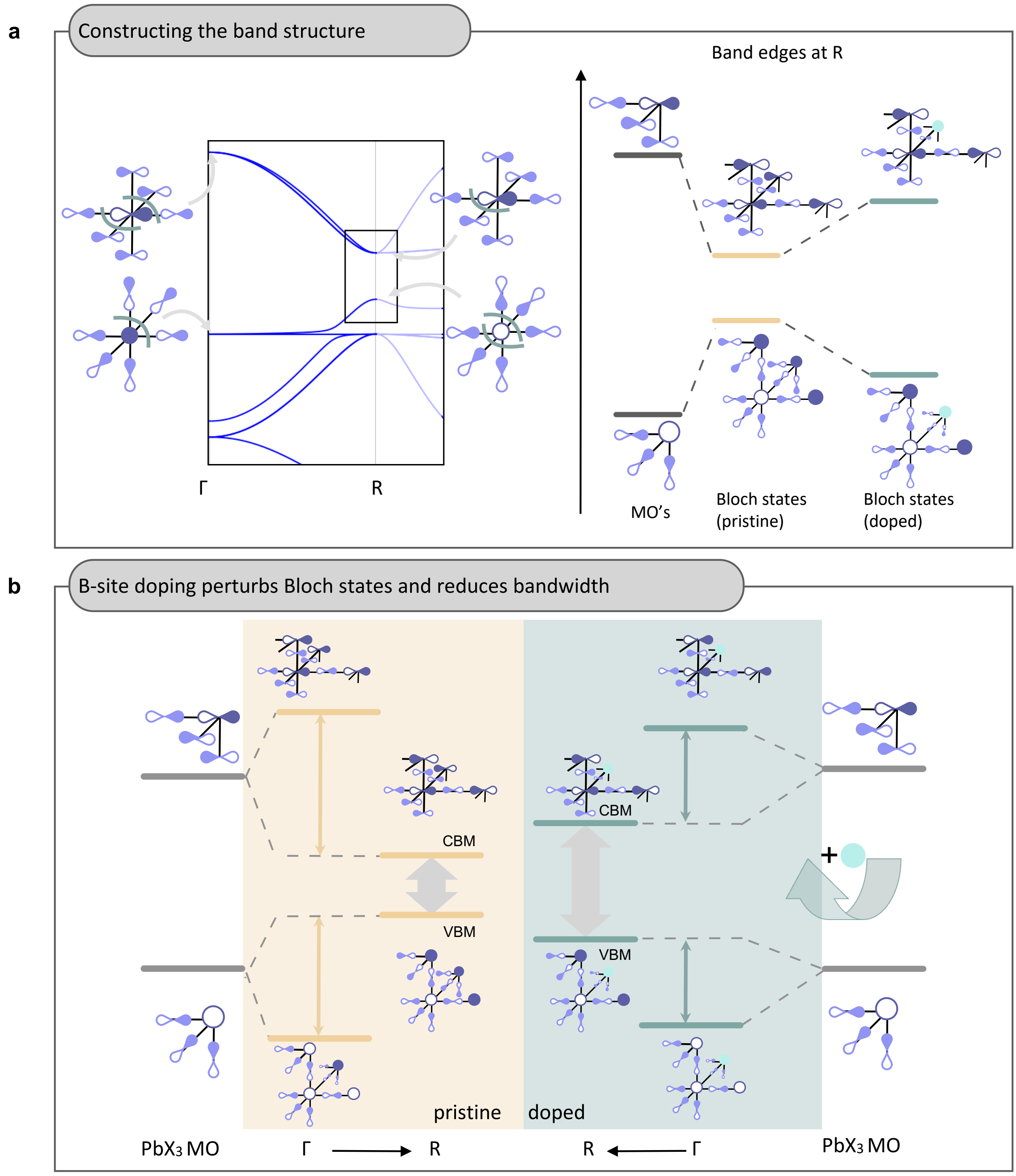

Supporting Figure S14: a, Left: Qualitative illustration of the band structure and the corresponding octahedral crystal orbitals for the VBM and CBM (both at the R-point), as well as the crystal orbitals of the lowest energy level in the valence band and the highest energy level in the conduction band (both at the Γ-point). Here it becomes clear that the bandwidth of the valence and conduction bands is determined by the bonding and antibonding nature of the Bloch states with different phase factors. Right: Schematic illustration of the band formation at the R-point starting from the molecular orbitals of the PbX_3 unit, and the effect of breaking periodicity by introducing a B-site dopant (blue circle). **b,** Left: Schematic representation of the Bloch states formed at the Γ-point and the R-point to build the valence and the conduction band, respectively. The yellow double arrows highlight the bandwidth of the bands, whereas the grey double arrow shows the direct electronic band gap.

Right: Schematic representation of the same states, now showing the relative change of the electronic energy when a B-site dopant is incorporated. It becomes clear that 1) the band gap widens because of stabilization and destabilization of the VBM and the CBM, respectively, and that 2) the bandwidth for both energy bands is reduced upon doping.

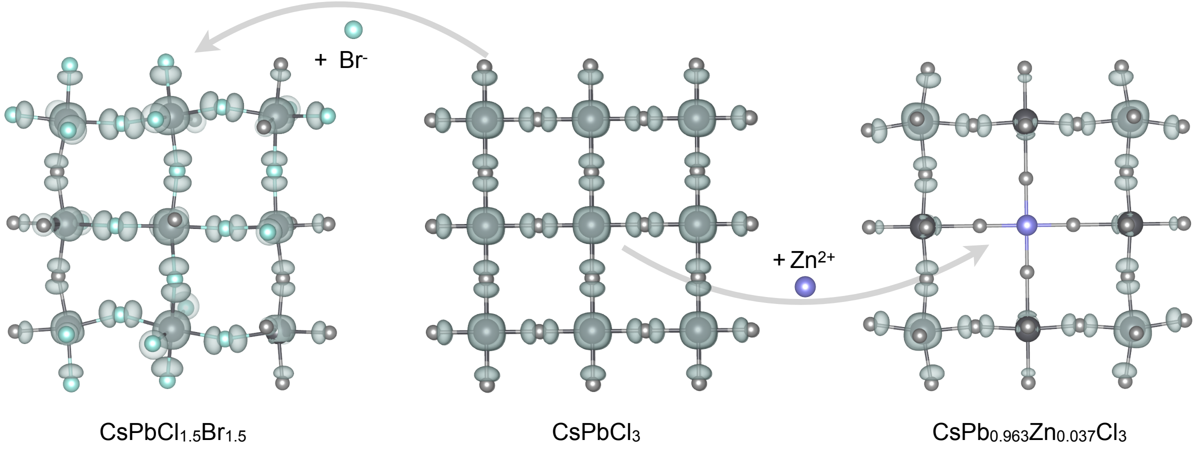

Supporting Figure S15: Comparison between the VBM charge density of the pristine $CsPbCl_3$, the mixed halide $CsPbBr_{1.5}Cl_{1.5}$ and the Zn-doped $CsPbCl_3$. It becomes evident that the impact of B-site doping (here shown for Zn) on the charge localization is much larger than that of the halide mixing, proving the rationale that B-site doping breaks periodicity while halide alloying does not. The charge densities were calculated using the PBEsol functional with spin-orbit coupling.

References

1. De Mello, J. C., Wittmann, H. F. & Friend, R. H. An improved experimental determination of external photoluminescence quantum efficiency. *Adv. Mater.* **9**, 230–232 (1997).
2. Makarov, N. S. *et al.* Spectral and Dynamical Properties of Single Excitons, Biexcitons, and Trions in Cesium-Lead-Halide Perovskite Quantum Dots. *Nano Lett.* **16**, 2349–2362 (2016).
3. Motti, S. G. *et al.* $CsPbBr_3$ Nanocrystal Films: Deviations from Bulk Vibrational and Optoelectronic Properties. *Adv. Funct. Mater.* **1909904**, 1–9 (2020).
4. Donegá, C. D. M. & Koole, R. Size dependence of the spontaneous emission rate and absorption cross section of CdSe and CdTe quantum dots. *J. Phys. Chem. C* **113**, 6511–6520 (2009).
5. Granados Del Águila, A., Groeneveld, E., Maan, J. C., De Mello Donegá, C. & Christianen, P. C. M. Effect of Electron-Hole Overlap and Exchange Interaction on Exciton Radiative Lifetimes of CdTe/CdSe Heteronanocrystals. *ACS Nano* **10**, 4102–4110 (2016).
6. Gong, K., Martin, J. E., Shea-Rohwer, L. E., Lu, P. & Kelley, D. F. Radiative lifetimes of zincblende CdSe/CdS quantum dots. *J. Phys. Chem. C* **119**, 2231–2238 (2015).
7. Morgan, D. P. & Kelley, D. F. Exciton localization and radiative lifetimes in CdSe Nanoplatelets. *J. Phys. Chem. C* **123**, 18665–18675 (2019).

8. Efros, A. L. & Rodina, A. V. Band-edge absorption and luminescence of nonspherical nanometer-size crystals. *Phys. Rev. B* **47**, 10005–10007 (1993).
9. Becker, M. A. *et al.* Bright triplet excitons in caesium lead halide perovskites. *Nature* **553**, 189–193 (2018).
10. Efros, A. L. Luminescence polarization of CdSe microcrystals. *Phys. Rev. B* **46**, 7448–7458 (1992).
11. Efros, A. L. *et al.* Band-edge exciton in quantum dots of semiconductors with a degenerate valence band: Dark and bright exciton states. *Phys. Rev. B - Condens. Matter Mater. Phys.* **54**, 4843–4856 (1996).
12. Feldmann, S. *et al.* Charge Carrier Localization in Doped Perovskite Nanocrystals Enhances Radiative Recombination. *J. Am. Chem. Soc.* **143**, 8647–8653 (2021).
13. Kresse, G. & Furthmüller, J. Efficient iterative schemes for ab initio total-energy calculations using a plane-wave basis set. *Phys. Rev. B* **54**, 11169–11186 (1996).
14. Kresse, G. & Furthmüller, J. Efficiency of ab-initio total energy calculations for metals and semiconductors using a plane-wave basis set. *Comput. Mater. Sci.* **6**, 15–50 (1996).
15. Blöchl, P. E. Projector augmented-wave method. *Phys. Rev. B* **50**, 17953–17979 (1994).
16. Kresse, G. & Joubert, D. From ultrasoft pseudopotentials to the projector augmented-wave method. *Phys. Rev. B* **59**, 1758–1775 (1999).
17. Koelling, D. D. & Harmon, B. N. A technique for relativistic spin-polarised calculations. *J. Phys. C Solid State Phys.* **10**, 3107–3114 (1977).
18. Perdew, J. P. *et al.* Restoring the Density-Gradient Expansion for Exchange in Solids and Surfaces. *Phys. Rev. Lett.* **100**, 136406 (2008).
19. Monkhorst, H. J. & Pack, J. D. Special points for Brillouin-zone integrations. *Phys. Rev. B* **13**, 5188–5192 (1976).
20. Adamo, C. & Barone, V. Toward reliable density functional methods without adjustable parameters: The PBE0 model. *J. Chem. Phys.* **110**, 6158–6170 (1999).
21. Perdew, J. P., Ernzerhof, M. & Burke, K. Rationale for mixing exact exchange with density functional approximations. *J. Chem. Phys.* **105**, 9982–9985 (1996).
22. Kang, J. & Wang, L.-W. High Defect Tolerance in Lead Halide Perovskite $CsPbBr_3$. *J. Phys. Chem. Lett.* **8**, 489–493 (2017).
23. Yin, W.-J., Yan, Y. & Wei, S.-H. Anomalous Alloy Properties in Mixed Halide Perovskites. *J. Phys. Chem. Lett.* **5**, 3625–3631 (2014).
24. Muhammad, Z. *et al.* Tunable relativistic quasiparticle electronic and excitonic behavior of the $FAPb(I_{1-x}Br_x)_3$ alloy. *Phys. Chem. Chem. Phys.* **22**, 11943–11955 (2020).
25. Chen, Y. *et al.* Optoelectronic Properties of Mixed Iodide–Bromide Perovskites from First-Principles Computational Modeling and Experiment. *J. Phys. Chem. Lett.* **13**, 4184–4192 (2022).
26. Diez-Cabanes, V., Even, J., Beljonne, D. & Quarti, C. Electronic Structure and Optical Properties of Mixed Iodine/Bromine Lead Perovskites. To Mix or Not to Mix? *Adv. Opt. Mater.* **9**, 2001832 (2021).
